# SARS-CoV-2 triggers an NF-kB-driven proliferative response in epididymal clear cells of K18-hACE2 mice

**DOI:** 10.1530/RAF-25-0197

**Published:** 2026-05-28

**Authors:** A A S Da Silva, O S Akinsomisoye, M A Battistone, P S Cerri, E Sasso-Cerri

**Affiliations:** ^1^ Department of Morphology and Genetics, Federal University of São Paulo, São Paulo, Brazil; ^2^ Department of Morphology, Genetics, Orthodontics and Pediatric Dentistry, São Paulo State University (Unesp) School of Dentistry, Araraquara, Brazil; ^3^ Faculty of Basic Medical Sciences, Obafemi Awolowo University, Ile Ife, Nigeria; ^4^ Program in Membrane Biology, Nephrology Division, Department of Medicine, Massachusetts General Hospital and Harvard Medical School, Boston, Massachusetts, USA

**Keywords:** SARS-CoV-2, epididymis, clear cells, proton-secreting cells, mitotic activity

## Abstract

**Graphical Abstract:**

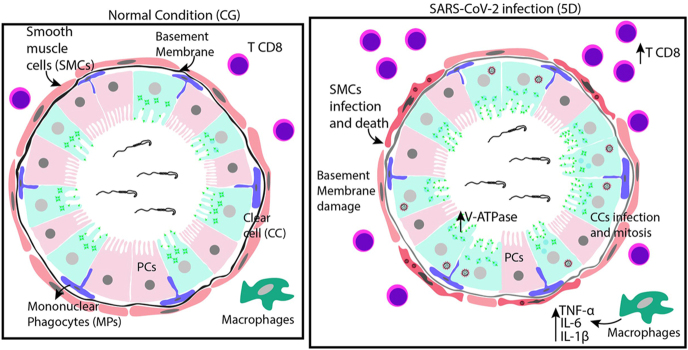

**Abstract:**

We examined SARS-CoV-2 infection in the epididymis of K18-hACE2 mice, focusing on the cauda region. Thirty mice were divided into control (CG), 2-day infection (2D), and 5-day infection (5D) groups. The animals were intranasally infected with SARS-CoV-2. The 5D group showed a significant increase in IL-6, TNF-a, and IL-1b, high mRNA levels of *Ifna*, *Ifnb*, and *Ifng*, and infiltration of CD8^+^ T cells. SARS-CoV-2 directly infects smooth muscle cells, leading to atrophy of the smooth muscle layer. The infection impaired the basement membrane and downregulated blood–epididymis barrier-related genes *Ocln* and *Jam-a*. Clear cells (CCs) exhibited increased apical V-ATPase and immunoexpression of hACE2, spike, and nucleocapsid. Interestingly, the infected groups showed a greater number of juxtaposed CCs with PCNA- and Ki67-positive nuclei. The findings also revealed a newly identified NF-kB-driven inflammatory-proliferative response in CCs. By combining *in vivo* and *in silico* analysis of a parallel LPS-stimulation RNA-seq dataset, we uncovered significant co-enrichment of the TNF-a–NF-kB pathway alongside cell cycle pathways (G2/M, E2F). The protein–protein interaction network identified key nodes linking the inflammatory NF-kB pathway to the cell cycle machinery. This *in silico* model was confirmed *in vivo*, demonstrating colocalization of phosphorylated NF-kB and PCNA in the CC rows. Alterations in spermatozoa, including decreased PNA labeling, ultrastructural changes, and reduced *Crisp1* mRNA, were also observed. Therefore, SARS-CoV-2 infection causes structural and functional changes in the epididymal cauda, leading to smooth muscle cell death and basement membrane disruption. Overall, the results shows that SARS-CoV-2 infection induces dysfunction in the cauda, and points CCs as players in the epididymal immune response.

**Lay summary:**

SARS-CoV-2, the virus that causes COVID-19, can reach many organs beyond the lungs, including the male reproductive tract. We studied how this virus affects epididymis, a sperm-carrying tube where sperm mature and are stored, in a genetically modified mouse model that expresses the human ACE2 receptor, which allows SARS-CoV-2 infection. We demonstrated that infection triggers a strong local inflammatory response and impairs the muscular wall and spermatozoa. The virus also infects specialized clear cells that control the epididymal fluid pH where sperm are stored. Following infection, clear cell groups showed proliferation and possible acidification of the sperm storage microenvironment. All these combined changes suggest that the normal environment required for sperm maturation is disrupted. Our findings raise concern that severe COVID-19 may have lasting effects on male fertility and highlight the epididymis as an important but overlooked target organ of this infection.

## Introduction

COVID-19 may affect the male reproductive system through various mechanisms. The virus can potentially invade the genital tract due to high levels of ACE2 and TMPRSS2 receptors in testicular cells (Sheikhzadeh Hesari *et al.* 2021). While most studies have found no SARS-CoV-2 in semen, one study reported its presence in human semen (He *et al.* 2021), and SARS-CoV-2 particles have also been detected in sperm cells (Hallak *et al.* 2024). COVID-19 may impact male fertility through direct viral effects, secondary inflammatory responses, and cytokine storms (Ardestani Zadeh & Arab 2021). Testicular spermatogenic dysfunction has been observed in some patients (He *et al.* 2021). Impaired semen quality has been reported in moderate infections compared to mild cases and healthy controls, possibly due to fever and inflammation (He *et al.* 2021). In addition, testes are vulnerable to SARS-CoV-2, which can cause testicular dysfunctions (Duarte-Neto *et al.* 2021, de Oliveira *et al.* 2025), impairing steroidogenesis (de Oliveira *et al.* 2025) and spermatogenesis (Hallak *et al.* 2024, de Oliveira *et al.* 2026), leading to male infertility (Duarte-Neto *et al.* 2021).

Recent studies have investigated the link between SARS-CoV-2 infection and conditions such as epididymitis, orchitis, and epididymo-orchitis (EO) in pediatric patients. A systematic review identified six cases of SARS-CoV-2-related EO in children, with four of these patients also diagnosed with multisystem inflammatory syndrome (Inouye *et al.* 2023). Another case series reported three children with acute EO following PCR-confirmed SARS-CoV-2 infection (Hoffmann & Gopal 2024). Although SARS-CoV-2 has been shown to affect the testis and epididymis in adults, studies using k18-hACE2 transgenic mice, which express the human angiotensin converting enzyme 2 (hACE2), have demonstrated that SARS-CoV-2 can infect both the testis (de Oliveira *et al.* 2025, 2026) and epididymis (da Silva *et al.* 2025*b*), causing significant damage to these organs and triggering immune responses in Leydig cells, Sertoli cells, and CCs.

The blood–epididymis barrier (BEB) plays a vital role in male fertility by creating a specialized luminal environment for sperm maturation (Gregory & Cyr 2014). The BEB is composed of three main components: anatomical (tight junctions), physiological (transporters), and immunological (immune privilege) (Mital *et al.* 2011). It controls the exchange of molecules between the blood and the epididymal lumen, protecting sperm from immune responses (Mital *et al.* 2011, Gregory & Cyr 2014).

Proton-secreting CCs help regulate luminal pH by recruiting the proton pump V-ATPase, which is found only in these cells in the epididymis (Breton & Brown 2013). Recent research shows that these cells also play a role in the epididymal immune response during lipopolysaccharide (LPS) stimulation (Battistone *et al.* 2019*a, b*, da Silva *et al.* 2025*a*), and viral infections (da Silva *et al.* 2025*b*).

CD8 T cells play a vital role in fighting viral infections, especially respiratory viruses (Schmidt & Varga 2018). These cells are crucial for clearing acute infections and establishing long-term immunity (Cox *et al.* 2013). During viral infections, CD8 T cells undergo complex differentiation, resulting in the formation of memory CD8 T cells (Wherry & Ahmed 2004). Cytokines greatly influence CD8 T cell responses, serving as immune warning signals and supporting cell survival (Cox *et al.* 2013). CD8 T cell infiltration was also observed in the epididymis after regulatory T cell (Treg) depletion (Elizagaray *et al.* 2025). Cytokines are key players in both protective and harmful immune responses during viral infections (Wack *et al.* 2011). In epididymal infections, pro-inflammatory cytokines like interleukin-1a (IL-1a), IL-1b, and IL-4 are significantly elevated (Turner *et al.* 2011). Uropathogenic *E. coli* infection can lead to fibrosis and ductal blockage in the epididymis, causing increased levels of fibrotic markers and cytokines such as activin A, tumor necrosis factor-alpha (TNF-a), and IL-6 (Michel *et al.* 2016). Likewise, Zika virus (ZIKV) infection can cause inflammation in the testis and epididymis, resulting in testicular damage and potential male infertility in mice, through innate immune responses in diverse cell types that produce pro-inflammatory cytokines (Ma *et al.* 2016).

Mouse epididymal epithelial cells express various viral sensors that initiate innate antiviral responses when activated by viral components (Zhu *et al.* 2015). Hepatitis E virus infection in mice causes testicular damage, impairs sperm quality, and disrupts the blood–testis barrier (Situ *et al.* 2020). ZIKV infection can cause significant damage to the male reproductive system in mice. Studies have shown that ZIKV can replicate efficiently in testes and epididymis, leading to inflammation, tissue damage, and potential infertility (Ma *et al.* 2016, Sheng *et al.* 2021). In the epididymis, ZIKV disrupts barrier structure, absorption/secretion functions, and impairs the microenvironment for sperm maturation (Sheng *et al.* 2021). Inflammatory responses, including increased production of cytokines IL-6 and IL-28, are observed in infected epididymal tissues (Sheng *et al.* 2021).

In our previous study using the SARS-CoV-2 infection model, we showed that this virus infects epididymal cells and causes epithelial damage. We also demonstrated that epididymal regions respond differently to infection, with the cauda being more susceptible than other regions (da Silva *et al.* 2025*b*). Thus, in this study, we evaluated the expression of cytokines, CD8 cells infiltration, and the integrity of the basement membrane and smooth muscle cells layer of the cauda region. Since CCs play a role in the epididymal immune response to SARS-CoV-2, the immunolocalization of V-ATPase and NF-kB, along with the proliferative activity of these cells, was also evaluated.

## Materials and methods

### Animal procedures and sample collection

For this study, both right and left epididymides from transgenic mice, belonging to infected and control groups, were kindly provided by Dr Thiago Mattar Cunha of São Paulo University (Ribeirão Preto, Brazil). A detailed description of the animal husbandry and experimental design is outlined below.

All experimental protocols and animal handling received prior approval from the São Paulo State University’s Ethical Committee for Animal Research (UNESP/FOAr, Brazil; 03/2022). The study utilized thirty 12-week-old male mice (*Mus musculus*), genetically modified (K18-hACE2) from C57BL/6 background (McCray *et al.* 2007). The animals were housed in the virology research center FMRP/USP under standard conditions, such as a 12 h light:12 h darkness cycle, regulated temperature (23 ± 2°C), and humidity levels between 65 and 75%. Food and water were available *ad libitum*.

Thirty animals were allocated into three groups of 10 animals each (*n* = 10/group): a control group (CG), 2-day infection group (2D), and a 5-day infection group (5D). The procedures were conducted in a biosafety level 3 facility. Animals in the 2D and 5D groups were inoculated intranasally with 40 µL of 5 × 10^4^ plaque-forming units (PFU). Concurrently, the CG animals received an equal volume (40 μL) of Dulbecco’s modified Eagle’s medium (DMEM) via the same route of administration. Animal weight and clinical manifestations were monitored daily throughout the experiment, starting from a pre-infection baseline.

Upon completion of the treatment period, the mice were anesthetized with ketamine hydrochloride (80 mg/kg b.w.) and xylazine hydrochloride (8 mg/kg b.w.). For each group, the cauda region of the right epididymis was immersed in fixative solution for histological examination. The cauda region of the left epididymis was immersed in 1 mL of RNA Keeper and storage at −80^°^ for RT-PCR analysis.

### Light microscopy

For light microscopy, the epididymides were fixed for 48 h in a 4% formaldehyde solution, prepared from paraformaldehyde and buffered at pH 7.4 with 0.1M sodium phosphate. Following fixation, the tissues were dehydrated through a graded series of alcohol and embedded in both paraffin and glycol methacrylate according to Cerri & Sasso-Cerri (2003), using a HistoResin kit (Leica, Germany).

The 5 μm-thick paraffin sections were stained with Masson’s trichrome or hematoxylin and eosin (H.E.). Some sections were also mounted on silanized slides for TUNEL (Terminal deoxynucleotidyl transferase dUTP Nick End Labeling) and immunofluorescence analyses, as described later. Similarly, 4 μm-thick sections from HistoResin blocks were cut using a Leica RM2255 microtome and stained with H.E. for morphological assessment, following the protocol by Cerri & Sasso-Cerri (2003).

### Transmission electron microscopy (TEM)

For ultrastructural analysis, small fragments from the epididymal cauda were initially fixed for 16 h in a mixture of 4% formaldehyde and 5% glutaraldehyde, buffered to pH 7.2 with 0.1 M sodium cacodylate. The samples were then washed in the same buffer before being post-fixed for 1 h in sodium cacodylate-buffered 1% osmium tetroxide at pH 7.2. Subsequently, the tissue was stained *en bloc* with 2% aqueous uranyl acetate for 1.5 h, dehydrated in a graded ethanol series, treated with propylene oxide, and embedded in Araldite resin (da Silva *et al.* 2023).

To identify regions of interest, semithin sections were first cut with glass knives on an ultramicrotome, stained with 1% toluidine blue, and observed under a light microscope. Ultrathin sections were collected on grids, contrasted with alcoholic 2% uranyl acetate and lead citrate, and observed using a Tecnai G2 Spirit transmission electron microscope.

### Morphological and quantitative analyses

Digital images for analysis were acquired with a DP-71 Olympus (Olympus, Japan) camera attached to a BX-51 Olympus light microscope (Olympus, Japan). All morphometric measurements were conducted using the Image-Pro-Express 6.0 software system.

### Smooth muscle layer thickness

The thickness of the smooth muscle cell layer was measured using four H.E.-stained sections from each animal. Analysis was limited to standardized duct regions in the distal cauda, as described by Johnston *et al.* (2005). For each section, images of 19 duct cross-sections were captured at ×40 magnification. In each duct section, two measurements of the muscular layer thickness surrounding opposite epithelial surfaces were recorded, and the average for each measurement was calculated (da Silva *et al.* 2023).

### Rows of CCs

The length of the basal epithelial surface with more than three juxtaposed CCs forming a row was measured. Ten images from 2 non-serial epididymal sections stained with H.E. at ×40, resulting in a total of 20 images of each animal from CG, 2D, and 5D, were used. In each image, the epithelial perimeter of each duct section and the CC rows were measured using Image-Pro Express 6.0 software. Then, the total CC rows were divided by the total epithelial perimeter, and the CC rows length/epithelial perimeter (μm) was obtained.

### Gomori method for reticular fibers

To identify the reticular fibers in the epididymal cauda, a silver impregnation method was used. As previously described (Gomori 1937), after deparaffinization, epididymal sections were impregnated with ammoniacal silver nitrate and the reaction was revealed with gold chloride. After staining, the sections were dehydrated and cleared in xylene, then mounted with the resinous mounting medium Permount™.

### Aniline blue staining

The acid aniline blue staining method (Terquem & Dadoune 1983) was adapted and used as a marker of histone in the sperm cells. After deparaffinization with hexane and hydration, the sections were stained with 5% aniline blue at pH 3.5 for 5 min. In this way, spermatozoa nuclei with mature chromatin are weakly stained, whereas immature sperm chromatin is strongly stained in blue.

### TUNEL assay

Cell death was identified using the TUNEL assay following the protocol of the ApopTag® Peroxidase *in situ* Apoptosis Detection Kit (Merck, Germany) and Sasso-Cerri & Cerri (2008). The sections were treated with proteinase K, then endogenous peroxidase activity was quenched with hydrogen peroxide. The tissue was incubated in an equilibration buffer for 30 min at room temperature, followed by a 1 h incubation at 37°C with terminal deoxynucleotidyl transferase (TdT). After incubation with an anti-digoxigenin-peroxidase antibody, the reaction was visualized with 0.06% 3,3′-diaminobenzidine (DAB). Sections were counterstained with Carazzi’s hematoxylin before dehydration and mounting. For negative controls, the TdT enzyme was omitted from the incubation solution. Testicular sections from mice served as positive controls.

### Immunostaining protocols

#### Immunofluorescence or immunohistochemistry reactions

For antigen retrieval, the sections were immersed in 0.001 M sodium citrate buffer (pH 6.0) or 0.1 M TRIS/EDTA buffer (pH 9.0) and heated in a Decloaking Chamber™ NxGen (BioCare Medical, USA) for 30 min at 90°C. Non-specific antibody binding was then blocked by incubating the sections for 20 min in 1% BSA. The sections were incubated overnight at 4°C with the following primary antibodies: rabbit anti-TNF-a polyclonal antibody (RRID: AB_10891701, 1:500, Boster Biological Technology, USA; code: PA1079, lot:0101812Da45079125), rabbit anti-IL-1b polyclonal antibody (RRID: AB_308765, 1:400, Abcam, USA; code: ab9722, lot:GR3175415-1), goat anti-IL-6 polyclonal antibody (RRID: AB_2127470, 1:50, Santa Cruz Biotechnology, USA Cat# sc-1265), rabbit anti-claudin-1 monoclonal antibody (ab307692, 1:200, Abcam, USA), mouse anti-CD8 (OX8) monoclonal antibody (sc-53063, conjugate with Alexa Fluor 488, Oregon, USA), and rabbit anti-V-ATPase polyclonal antibody (0.3 μg/mL; Abcam; ab73404).

The sections were incubated for 1 h at room temperature in the dark with the corresponding secondary antibodies: Alexa Fluor 488 goat anti-mouse IgG (1:1,000), Alexa Fluor 647 goat anti-rabbit IgG (1:1,000), or Alexa Fluor 647 donkey anti-goat antibody IgG (1:1,000). DAPI or propidium iodide (PI) was used as a nuclear labeling. Negative controls were performed by replacing the primary antibody with non-immune serum (Supplementary Fig. 1 (see section on Supplementary materials given at the end of the article)).

#### Double immunolabeling reactions

Immunofluorescence reactions were performed to investigate the colocalization of PCNA + V-ATPase, Ki67 + V-ATPase, hACE2 + V-ATPase, Spike + V-ATPase, nucleocapsid + V-ATPase in CCs, Spike + Actin and nucleocapsid + Actin in the muscular layer, and phosphorylated NF-kB (NF-kB-p) + PCNA. For antigen retrieval, the sections were immersed in sodium citrate buffer (pH 6.0) or 0.1 M TRIS/EDTA buffer (pH 9.0) and heated in a Decloaking Chamber™ NxGen (BioCare Medical) at 90°C for 30 min, and incubated for 20 min in 1% BSA to avoid non-specific antibody binding. Then, the sections were incubated overnight at 4°C with mouse anti-PCNA monoclonal antibody (1:200; BioCare; CM152), rabbit anti-SARS-CoV-2 spike protein S1 monoclonal antibody (1:50, Thermo Fisher Scientific, USA, code: MA5-36247), anti-SARS-CoV-2 nucleocapsid protein monoclonal antibody (1:1,000; EPR24334-118; Abcam, UK; ab271180), rabbit anti-Ki67 antibody (SP6) (1:100, Abcam, Ab16667), mouse anti-hACE2 (AC18Z), monoclonal antibody (1:500, Santa Cruz Biotechnology; code: sc-73668), and rabbit anti-phospho-NF-kB p65 (Ser536) (1:100, Cell Signaling, USA, #3031). After washing, the sections were incubated with the following secondary antibodies: Alexa Fluor 594 anti-mouse (1:1,000, Abcam, Ab150116), Alexa Fluor^®^488 anti-rabbit IgG antibody (1:1,000; Molecular Probes^®^ by Life Technologies, USA, A11001, lot: 1664729), and Alexa Fluor^®^594 anti-rabbit IgG antibody (1:500; Invitrogen^®^ by Thermo Fisher Scientific, USA, code: R3117, lot: 2086924). After washing, the sections were incubated overnight at 4°C with rabbit anti-V-ATPase polyclonal antibody (0.3 μg/mL; Abcam; ab73404), rabbit anti-actin polyclonal antibody (1:1,000, RRID: AB_476693; Sigma-Aldrich, Germany; code: A2066), and mouse anti-PCNA monoclonal antibody (1:200; BioCare; CM152). After washing, the sections were incubated with Alexa Fluor 488 anti-rabbit antibody, Alexa Fluor^®^350 anti-rabbit antibody (1:1,000; Molecular Probes^®^ by Life Technologies, USA, A11069, lot: 1371039), and Alexa Fluor 594 anti-mouse antibody (1:1,000, Abcam, Ab150116). DAPI or propidium iodide (PI) was used as nuclear labeling.

#### Analysis of immunolabeled area

The measurement of the immunolabeled areas was performed in images acquired with a Leica DFC 550 camera attached to a Leica BM4000 B LED microscope and a Leica Application Suite (LAS 4.3) software. For IL-6, IL-1b, TNF-a, CD8, and V-ATPase markers, the immunolabeled area was measured across four non-consecutive sections per animal. The analysis was performed in a standardized total stromal or epithelial area, and the immunopositive area per μm^2^ of stroma or epithelium was calculated for each marker. To ensure consistency, parameters for threshold, color range, hue, and saturation were kept constant for each specific marker.

For the analysis of V-ATPase immunolocalization in the CCs, the total area of twelve CCs per animal was measured at ×100. In each CC, the V-ATPase-immunolabeled apical area was measured, and the V-ATPase-immunolabeled area/CC total area was calculated.

#### Peanut agglutinin (PNA) labeling

The acrosomal integrity was assessed using PNA labeling as a specific acrosome marker. After deparaffinization and hydration, the sections were submitted to antigen retrieval using 0.001 M sodium citrate buffer (pH 6.0) at 90°C for 30 min in a Decloaking Chamber™ NxGen (BioCare Medical). After washing with PBS, a permeabilization step was performed using PBS/T (0.1% Triton X-100) for 10 min and then the tissues were blocked by incubating the sections for 20 min in 1% BSA. The sections were incubated with 2 µg/mL (1:500) of Alexa Fluor^®^ 488-conjugate PNA (Molecular Probes^®^ by Life Technologies, USA, L21409) for 1 h at room temperature. DAPI was used as a nuclear labeling.

#### Analysis of PNA fluorescence area

The images were acquired by a Leica DFC 550 camera attached to a Leica BM4000 B LED microscope, and the analysis was performed using a Leica Application Suite (LAS 4.3) software. The acrosomal integrity was evaluated in two non-serial epididymal sections per animal. In each section, five epididymal duct sections in the distal cauda portion were captured at ×1,000, totalizing 10 sections per animal. In each duct section, the DAPI and PNA fluorescent areas were measured in a standardized luminal area. In the luminal region of each ductal section, the total DAPI-positive nuclear area of spermatozoa was obtained. In this same area of merged images (DAPI + PNA), the PNA-positive area was computed. Thus, the PNA-labeled area was normalized to the total nuclear area and expressed as PNA fluorescent area/DAPI fluorescent area.

#### Reverse transcription-quantitative PCR (RT-qPCR)

For gene expression analysis, the whole cauda was immediately stabilized in RNA Keeper stabilizing reagent upon collection and stored at −80°C. Total RNA was isolated from cauda regions using the Aurum Total RNA Mini Kit. The extracted RNA was then reverse transcribed into cDNA with high-capacity cDNA Reverse Transcription Kit (ThermoFisher, Cat: 4368814). Real-time quantitative PCR (RT-qPCR) was performed using a QuantStudio 3 instrument and Power Up SYBR Green Master Mix. Thermal cycling parameters were set according to the manufacturer’s protocol. Gene expression levels were quantified using the ΔCt method (ΔCt = (Ct target gene − Ct housekeeping gene b-actin)). The relative expression was then calculated using the log(2^–ΔΔCt^) formula, where –ΔΔCt represents the difference between the ΔCt of the epididymal 5D and the mean ΔCt of the control group. All primers were designed with the Primer3 program, using murine sequences obtained from the University of California, Santa Cruz (UCSC) Genome Browser (Untergasser *et al.* 2012) (Table 1).

**Table 1:** Oligonucleotide primers used in RT-qPCRs. All primers were supplied by Exxtend, Brazil.

Gene	Length (bp)	Tm °C	Sequence (5′-3′)	NCBI access number
*Infa*				NM_010502.2
	24	58	TTC​CTC​AGA​CTC​ATA​ACC​TGA​GGA	
	23	58	ATT​TGT​ACC​AGG​AGT​GTC​AAG​GC	
*Infb*				NM_010510.2
	21	60	GAC​GTG​GGA​GAT​GTC​CTC​AAC	
	22	60	GGT​ACC​TTT​GCA​CCC​TCC​AGT​A	
*Infg*				NM_00837.4
	22	56	CTT​GAA​AGA​CAA​TCA​GGC​CAT​C	
	22	54	CTT​GGC​AAT​ACT​CAT​GAA​TGC​A	
*Jam-a*				NM_172647.2
	21	60	GGT​CAG​CAT​CCA​CCT​CAC​TGT	
	20	60	AGG​TCA​GCA​CTG​CCC​TGT​TC	
*Ocln*				NM_001360539.1
	24	58	CCC​AGA​TTA​GAG​TCC​AAA​GTC​AGT	
	21	58	CGG​AAA​CCT​TAG​AGA​GAT​CCC	
*Crisp1*				NM_009638.3
	30	60	AAG​CCA​TCA​GAA​TTC​CAA​GAT​AGC​TCT​CAG	
	30	60	CTG​CTG​CAG​GTC​TGG​AAT​TAT​TTC​AAT​GTC	
*Foxi1*				NM_023907.4
	20	57	ACT​AAC​GCC​AGC​CCC​TTT​CT	
	19	59	AGG​TCG​CTG​GGC​AGT​AGC​T	
*Actb*				NM_007393.5
	20	57	GCTCGCTTCCTTTGTCCC	
	21	55	GAC​AAT​TGA​GAA​AGG​GCG​TG	

### Statistical analysis

Statistical analyses were performed using GraphPad Prism 8.4.3 software (GraphPad Software, USA). To examine whether the samples were normally distributed, the normality test (Shapiro–Wilk test) was applied. Differences between groups were analyzed by one-way ANOVA with Tukey’s post hoc test for multiple comparisons or Student’s* t-*test. The significance level was set as 0.05 (*P* < 0.05).

### Bioinformatics analysis

This involves reanalysis of bulk RNA-seq of epididymal CCs (da Silva *et al.* 2025*a*, GSE294713).

### Pathway analysis

Bulk RNA-seq of epididymal CCs was analyzed from three anatomical regions: IS/Caput, Corpus, and Cauda, after intravasal injection of Saline or LPS (25 μg) (*n* = 3 per region and condition). Importantly, raw counts were used as the primary input for voom to accurately calculate precision weights. Raw sequence reads were processed and mapped to the *Mus musculus* genome. To account for the mean–variance relationship of the RNA-seq data, count normalization and differential expression analysis were performed using the limma-voom pipeline (Law *et al.* 2014). Briefly, counts were transformed to log2-counts per million (log-CPM) and a precision weight was calculated for each observation to minimize the impact of low-count genes and technical noise.

Gene set enrichment analysis (GSEA) was performed using the fgsea package in R. Genes were ranked based on the *t*-statistic generated by the limma linear model to ensure that both the magnitude and direction of expression changes were represented. The analysis focused on the MSigDB Hallmark gene set collection. Pathways were considered significantly enriched when the false discovery rate (FDR) adjusted *P*-value was less than 0.05. For visualization of regional biological trends, the normalized enrichment score (NES) was plotted as a continuous heatmap across the IS/Caput, Corpus, and Cauda, with statistical significance (FDR < 0.05) indicated by asterisks. Detailed statistics for all pathways analyzed in the GSEA Hallmark collection, including NES and FDR values, are available in Supplementary Table 1.

### Software

Analyses were performed in R (4.4.3) with packages limma, edgeR, fgsea, GSEABase/fgsea gmt loader, ggplot2, dplyr, tidyr, viridis, and openxlsx (for outputs). GSEA used MSigDB (mouse symbol) GMTs loaded from local files.

### Heatmap generation

The heatmap was generated in R using the ComplexHeatmap package (Gu *et al.* 2016). The standardized expression matrix was visualized with the Heatmap function, applying a diverging color gradient (blue, white, red) created via colorRamp2 (c (−2, 0, 2)).

Hierarchical clustering of genes was performed based on Euclidean distance, while sample order was fixed to maintain experimental grouping. Annotation bars were added to indicate the epididymal region (IS.Caput vs Cauda) and pathway identity (NF-kB or cell division).

### Pathway crosstalk and protein–protein interaction (PPI) network analysis

To investigate the mechanistic crosstalk between the co-activated inflammatory and cell proliferation signaling pathways identified by GSEA (using the limma-voom results), protein–protein interaction (PPI) networks were constructed.

Leading-edge gene subsets, defined as the core subset of genes driving the enrichment score, from the significantly enriched Hallmark pathways (HALLMARK_TNFA_SIGNALING_VIA_NFKB, HALLMARK_E2F_TARGETS, and HALLMARK_G2M_CHECKPOINT) were extracted from the cauda region based on the respective LPS *versus* Saline comparisons. The gene list was combined into a single, consolidated list of unique *Mus musculus* gene symbols.

The gene list was submitted to the STRING (search tool for the retrieval of interacting genes/proteins) (https://string-db.org/, V12.0). A protein–protein interaction (PPI) network was constructed specifically for the species *Mus musculus*. Interactions displayed in the network were filtered using a minimum required interaction score of 0.700 and considering only interactions supported by experimental evidence, curated databases, and co-expression, to prioritize high-confidence biological interactions. The generated network was exported from STRING in Cytoscape 3.10.4. Network metrics were calculated using the integrated NetworkAnalyzer tool. The network was treated as undirected during the analysis. Betweenness centrality (BC) was used to identify key ‘nodes’ potentially connecting different functional modules. The top 10 nodes were defined as bridge nodes for subsequent comparative analysis.

A discrete mapping was applied to the fill color property based on the Pathway_Origin column (blue: NF-kB; green: cell division; red: Bridge). Unconnected nodes (singletons) were removed from the final layout. High-resolution images were exported as vector files (SVG).

### Single-cell RNA-seq analysis and pathway enrichment

Reanalysis of single-cell RNA-seq (Rinaldi *et al.* 2020, GSE145443): Raw single-cell RNA-seq data were processed using Seurat (v5) in R (v4.4.3). After data import, cells with low gene counts or high mitochondrial content were filtered out. Normalization and variance stabilization were performed using SCTransform, and highly variable genes were identified.

Principal component analysis (PCA) was applied for dimensionality reduction, followed by Uniform Manifold Approximation and Projection (UMAP) for visualization and unsupervised clustering. Cell identities were annotated based on known marker genes.

To enable pseudo-bulk differential expression analysis, cell-level counts were aggregated by cell type and sample. Pseudo-bulk count matrices were then analyzed using DESeq2 and edgeR, comparing ‘clear’ versus ‘principal’ cells. Genes were ranked by log_2_ fold change, and differential expression was considered significant at an adjusted *P*-value (Benjamini–Hochberg correction) < 0.05.

The top differentially expressed genes were visualized using dot plots generated with ggplot2. Functional enrichment of upregulated genes in CCs was evaluated using clusterProfiler (v4.10). Gene ontology (GO) and Kyoto Encyclopedia of Genes and Genomes (KEGG) enrichment analyses were conducted using mouse-specific annotations (org.Mm.e.g.db).

In addition, gene set enrichment analysis (GSEA) was performed using MSigDB Hallmark gene sets to identify enriched biological pathways (e.g. E2F targets, G2M checkpoint, mTORC1 signaling). Enrichment results were visualized with dot plots showing GeneRatio (x-axis), adjusted *P*-values (color), and gene counts (dot size).

All visualizations, including combined publication-ready panels, were generated in R using ggplot2, enrichplot, magick, and cowplot packages. All R scripts used for data processing and analysis are available from the corresponding author upon reasonable request.

## Results

### SARS-CoV-2 infection increases cytokines and T CD8^+^ cells and impairs the basement membrane in the epididymal cauda

**Figure 1:**
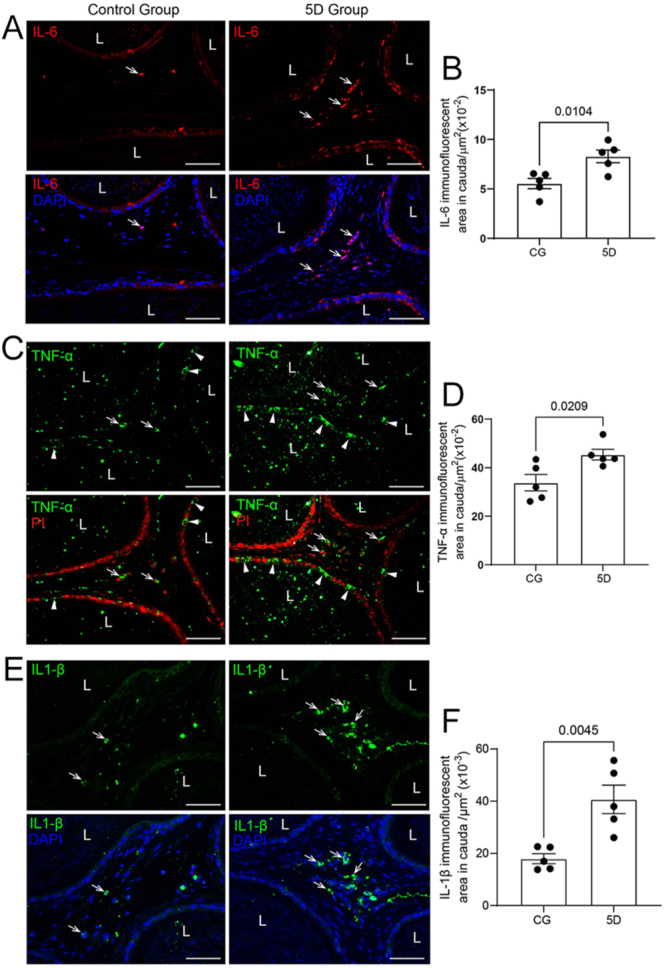
Pro-inflammatory cytokines expression in 5D cauda. (A) Immunofluorescence for IL-6 (red). (C) Immunofluorescence for TNF-a (green). (E) Immunofluorescence for IL-1b (green). Note the increased immunolabeling for IL-6, TNF-a, and IL-1b in the stroma of 5D when compared to CG (A, C, E, arrows). In panel C, TNF-positive CCs are indicated by arrowheads. L (lumen). In panels B, D, and F, the immunofluorescent areas for IL-6, TNF-a, and IL-1b increased in 5D when compared to CG. All analyses were performed in a standardized stromal area. Bars: 50 μm.

**Figure 2:**
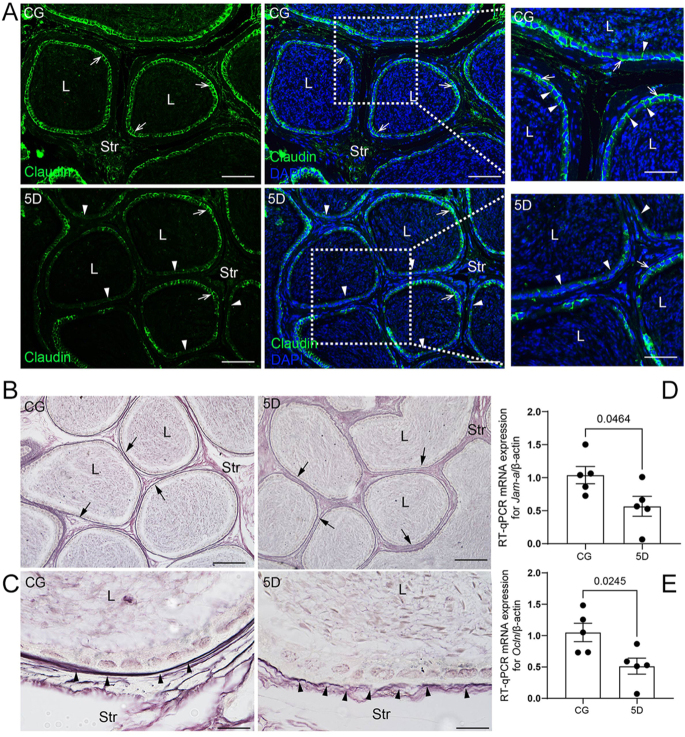
SARS-CoV-2 infection disrupts the BEB. (A) Immunofluorescence for claudin (green). In CG, note strong claudin immunolabeling in the epididymal duct (arrows), while in 5D, a weaker immunolabeling is observed (arrows). In the CG (inset), observe claudin at the base of epididymal cells (arrows) and between cells (arrowheads); however, in 5D (inset), note the weaker claudin immunolabeling between the epididymal cells (arrowheads). In panel B, Gomori’s silver impregnation shows reticular fibers. Note the presence of stained reticular fibers in the basement membrane of both CG and 5D (arrows); however, in 5D, the basement membrane (black arrowheads) stains weakly compared to the strong, continuous staining in CG (black arrowheads). In panel C, observe the weak, discontinuous basement membrane compared to CG (black arrowheads). In panels D and E, the mRNA levels of *Jam-a* and *Ocln* in whole cauda tissue are reduced in 5D compared to CG. Bars: panel A, 70 and 55 μm and panel B, 100 and 16 μm.

Following 5 days of SARS-CoV-2 infection, the immunoexpression of IL-6, TNF-a, and IL-1b increased in the cauda stroma compared to the control group (Fig. 1). The claudin analysis showed weak immunolabeling in the epididymal epithelium (Fig. 2A). Additionally, faint basement membrane positivity to silver staining (Fig. 2 and C) and downregulation of *Jam-a* and *Ocln* mRNA (Fig. 2D and E) were observed.

**Figure 3:**
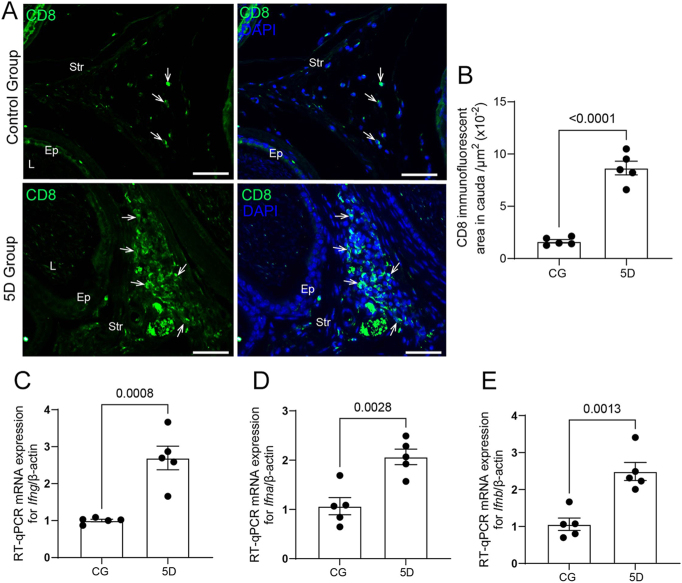
Recruitment of CD8 cells and expression of type I and type II interferons. (A) Immunofluorescence for CD8 (green). Note the abundance of CD8^+^ cells in the stroma of 5D compared to CG (arrows). (B) Increased CD8-immunolabeled area in a standardized stromal region of 5D compared to CG. In panels C, D, and E, observe the increased mRNA levels of *Infg*, *Infa*, and *Infb* in the epididymal cauda of animals from 5D compared to CG. Bars: 150 μm.

CD8 immunolabeling also increased in the stroma (Fig. 3A), and higher mRNA levels for *Infg*, *Infa*, and *Infb* were also detected in the epididymal cauda (Fig. 3C, D, E).

### SARS-CoV-2 infection impairs the muscular layer

**Figure 4:**
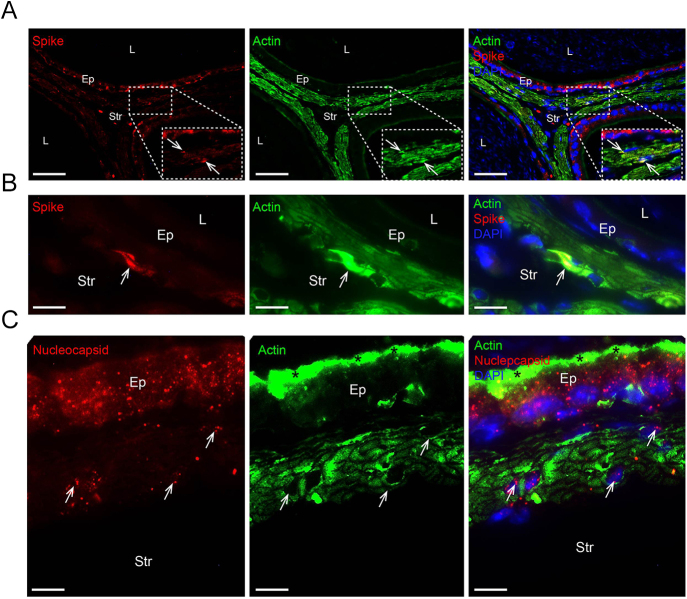
SARS-CoV-2 infects SMCs. Panels A and B show double immunolabeling for actin (green) and spike protein (red) in 5D. Panels A and B highlight the colocalization of spike protein and actin in some smooth muscle cells (arrows). Panel C displays double immunolabeling for actin (green) and nucleocapsid protein (red). Note the colocalization of nucleocapsid and actin in the smooth muscle cells (arrows). Actin-immunolabelled stereocilia (asterisks). Stroma (Str); epithelium (Ep); lumen (L). Bars: panel A, 50 μm; panel B, 15 μm; and panel C, 5 μm.

**Figure 5:**
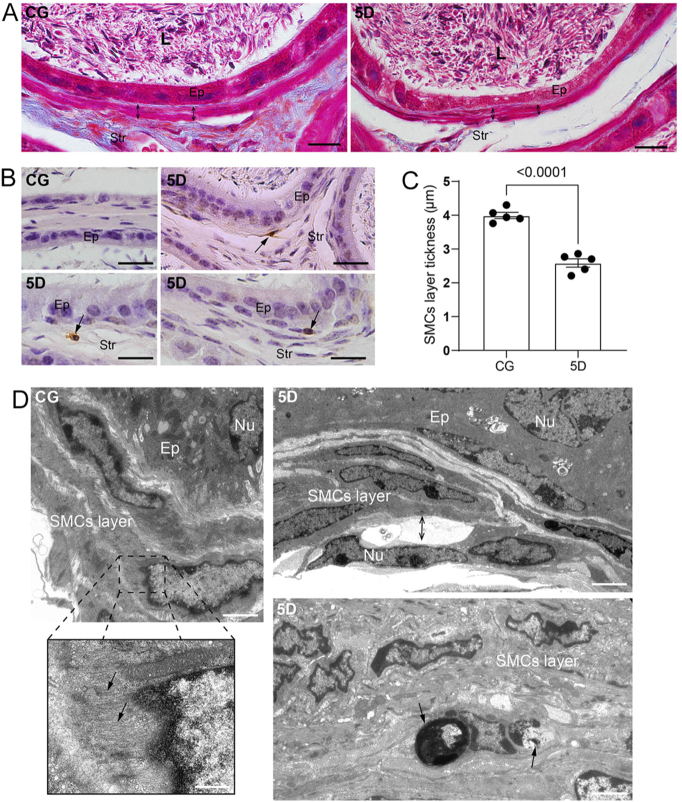
SARS-CoV-2 infection disrupts the SMC layer. Panel A shows epididymal sections stained with Masson’s trichrome, revealing a thinner smooth muscle layer in 5D compared to CG (double-ended arrows). In panel B, TUNEL-positive cells are seen in the muscular layer of 5D (arrows), while none are detected in CG. The smooth muscular layer is thinner in 5D compared to CG (C). In panel D, electron micrographs display actin filaments in the SMC cytoplasm of CG (arrows). In 5D, detached smooth muscle cells from the muscular layer (double-ended arrow) and apoptotic bodies in the SMC layer (arrows) are visible. Bars: panel A, 40 μm; panel B, 20 μm; and panel D, 800, 20, 750, and 550 nm.

After 5 days of infection, the smooth muscle cells were infected by SARS-CoV-2, as indicated by the detection of spike and nucleocapsid proteins in these cells, identified through double immunolabeling for actin + spike and actin + nucleocapsid (Fig. 4, panels A, B, and C). Considering the infection, we assessed the integrity of the smooth muscle layer. The infection decreased the thickness of the muscular layer (Fig. 5, panels A and C), and TUNEL-positive SMCs were observed in the cauda at 5D (Fig. 5, panel B). Ultrastructural analysis with TEM showed detachment of SMCs, clumps of condensed chromatin within the nuclei of these cells (Fig. 5, panel D), and the presence of apoptotic bodies in the muscular layer (Fig. 5, panel D).

### SARS-CoV-2 infects CCs and changes the immunolocalization of V-ATPase

**Figure 6:**
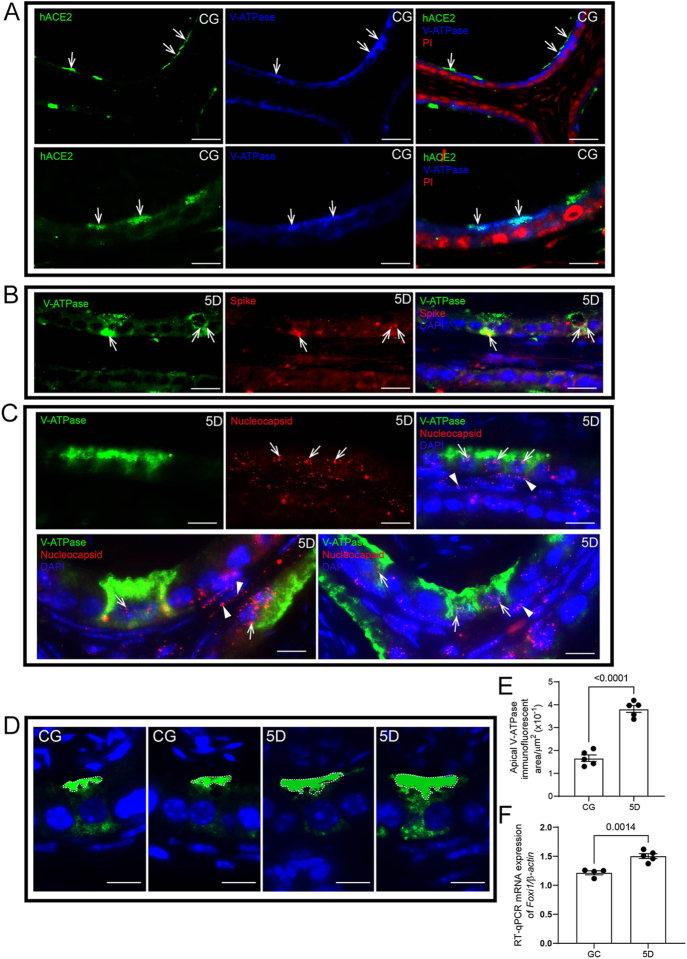
Infection of CCs and changes in V-ATPase immunoexpression pattern. Panel A shows double immunolabeling for hACE2 (green) and V-ATPase (blue). Note the colocalization of hACE2 and V-ATPase (arrows). Nuclear staining with propidium iodide (PI). In panel B, double immunolabeling for V-ATPase (green) and spike protein (red) reveals colocalization of V-ATPase and spike (arrows). The nucleus is stained with DAPI. Panel C, double immunolabeling for nucleocapsid (red) and V-ATPase (green), note the colocalization of nucleocapsid and V-ATPase (arrows) and the presence of nucleocapsid in the smooth muscle layer (arrowheads). Nuclear staining with DAPI. (D) strong V-ATPase immunolabeling in the apical region of CCs in 5D compared to CG (dashed line). In panel E, the apical V-ATPase immunofluorescent area increased in 5D compared to CG. The analysis was performed in a standardizedCC area. Panel F shows increased mRNA levels of *Foxi1* in 5D compared to CG. Bars: panel A, 50 and 10 μm; panel B, 12 μm; panel C, 10 μm; and panel D, 5 μm.

After 5 days of infection, double immunolabeling revealed V-ATPase and hACE2 in the CCs (Fig. 6A). In addition, SARS-CoV-2 spike protein and nucleocapsid were also detected in the V-ATPase-positive CCs (Fig. 6B and C). The SARS-CoV-2 infection changed the pattern of V-ATPase immunolocalization in CCs, becoming more concentrated in the apical region (Fig. 6D and E), and *Foxi1* mRNA was also upregulated 5 days after infection (Fig. 6F).

### The infection increases the mitotic activity in CCs and the formation of juxtaposed CC rows

**Figure 7:**
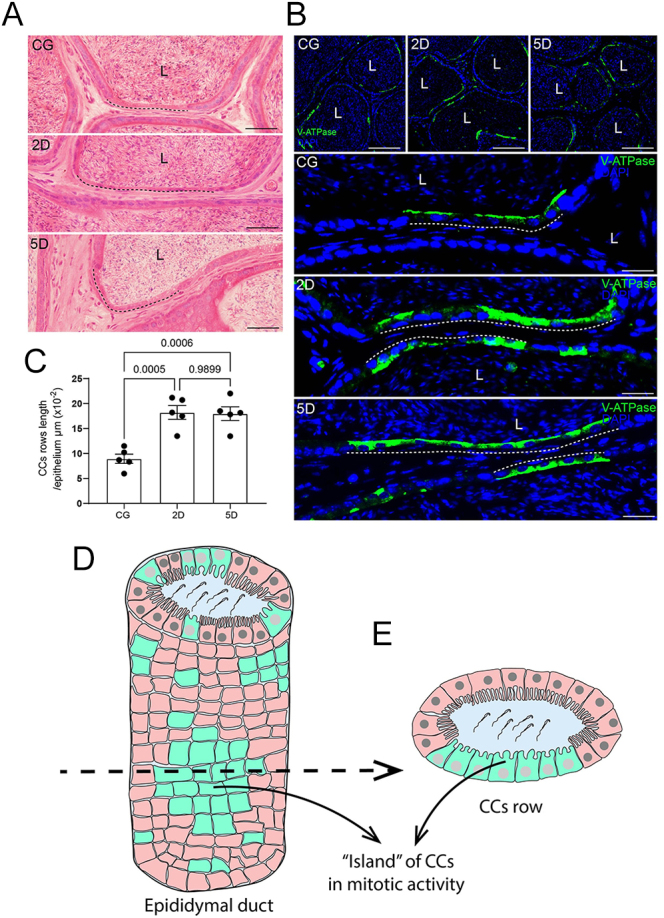
Increased CC rows in 2D and 5D. In panel A, H.E.-stained epididymal sections from CG, 2D, and 5D show CC rows (dashed lines) along the epithelium. (B) V-ATPase immunofluorescence (green) in CG, 2D, and 5D. Note the rows composed of juxtaposed CCs (dashed lines). In panel C, the lenght of CC rows increased in 2D and 5D compared to CG. Bars: panel A, 25 μm, and panel B, 83 and 16 μm. (D and E) A conceptualization of the CC row in the epithelium of the epididymal duct. (D) Side view of the epididymal duct showing the island of CCs in mitotic activity. (E) Epididymal cross-sectional view showing a CC row.

**Figure 8:**
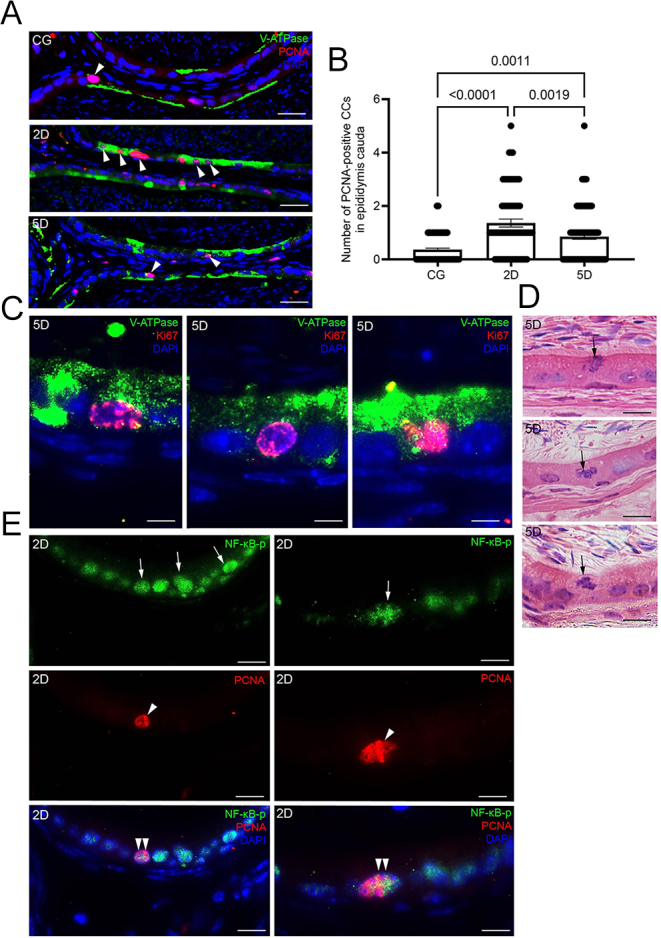
Proliferation of CCs during SARS-CoV-2 infection and colocalization of PCNA + NF-kB-p. Panel A shows double immunolabeling for V-ATPase (green) and PCNA (red). Note the colocalization of V-ATPase and PCNA in CCs (arrowheads). In panel B, the number of PCNA^+^ CCs increased in 2D and 5D compared to the control group; however, these cells decreased in 5D compared to 2D. In panel C, double immunolabeling for Ki67 (red) and V-ATPase (green) shows Ki67^+^ nuclei in the positive V-ATPase-labeled cells. In panel D (H.E. staining), cells in mitosis are observed in the cauda epithelium at 5D (arrows). In panel E, NF-kB-p immunolocalization in the nucleus of epithelial cells (green, arrows) and nuclear labeling of PCNA (red, arrowheads) are observed. Note the nuclear colocalization of NF-kB and PCNA in CCs (double arrowheads). Bars: panel A, 125 μm; panel B, 25 μm; panel C, 5 μm; panel D, 12 μm; and panel E, 10 μm.

In the H.E.-stained sections of epididymis from all groups, rows of closely packed CCs were observed in the cauda. CC rows were also observed in a C57BL/6 WT mouse (Supplementary Fig. 2). However, in 2 and 5 days of infection, long rows composed of several closely packed CCs were commonly seen (Fig. 7A). The basal epithelial surface measurement confirmed that the row length (composed of more than three juxtaposed CCs) increased significantly in 2 and 5 days compared to CG (Fig. 7B and C). The presence of these closely packed CC rows was verified by V-ATPase immunofluorescence (Fig. 7B). Double immunolabeling for V-ATPase + PCNA and V-ATPase + Ki67 showed colocalization of these proteins in CCs, indicating mitotic activity (Fig. 8A and C). CCs in various stages of mitotic division were also observed in the cauda from the 5D animals (Fig. 8D). The number of PCNA-positive CCs increased significantly in both 2 and 5 days compared to CG; however, this number was lower in 5 days compared to 2 days (Fig. 8B). The epididymal duct cross-sections showing several rows of CCs in mitotic activity indicate that these CCs are organized in ‘islands’ with mitotic potential along with the epithelial wall of the epididymis (Fig. 7D and E). Double immunolabeling for NF-kB-p and PCNA showed principal cells positive for NF-kB-p alone, and CCs positive for both NF-kB-p and PCNA (Fig. 8E).

### Spermatozoa alterations 5 days after SARS-CoV-2 infection

**Figure 9:**
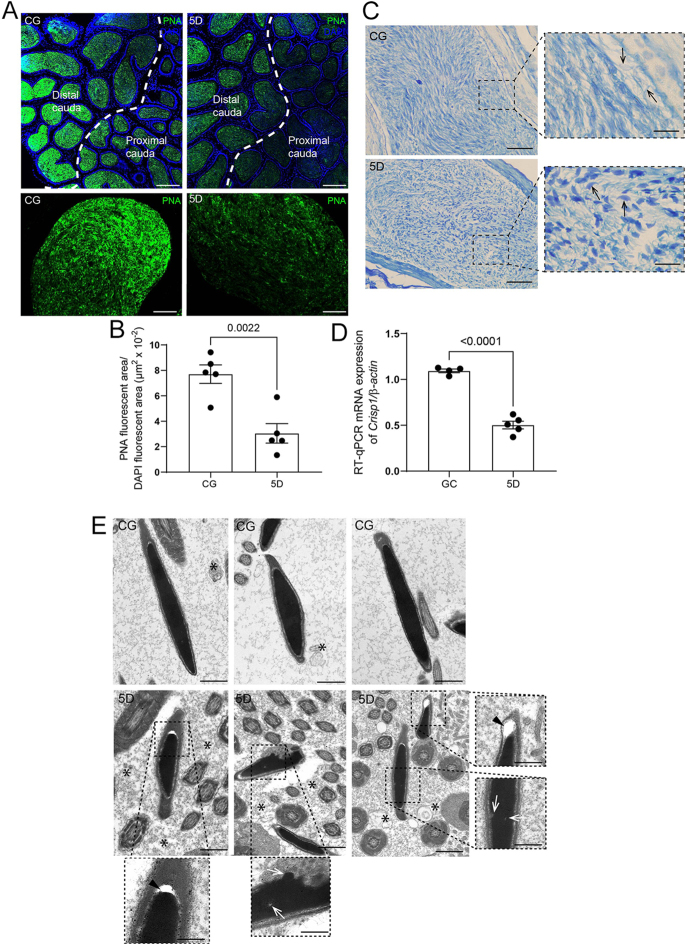
Changes in spermatozoa after 5 days of SARS-CoV-2 infection. In panel A, observe the weak PNA labeling in the proximal and distal cauda of 5D compared to CG. PNA fluorescence area decreases in the distal cauda of 5D versus CG (B). In panel C, acid aniline blue staining shows stained spermatozoa nuclei in the lumen of 5D, unlike the unstained nuclei in CG (inset, arrows). In panel D, *Crisp1* mRNA levels are reduced in 5D compared to CG. In panel E, spermatozoa nuclei display preserved ultrastructural integrity, with highly condensed, uniform chromatin and an intact acrosomal/nuclear interface. In 5D, electron-lucent dots appear in the chromatin (insets, white arrows), along with large sub-acrosomal spaces (inset, black arrowhead). The lumen of 5D is densely filled with cellular debris (asterisks) relative to CG. Bars: panel A, 70 and 15 μm; panel C, 15 and 11 μm; panel E, 17 nm; and insets, 5 nm.

The PNA labeling showed PNA-positive spermatozoa in the lumen of the epididymal cauda in animals from both the control and 5-day infected groups (Fig. 9A); however, weaker PNA labeling was observed in the 5-day infected group, along with a significant reduction in the PNA-positive area (Fig. 9B). In addition, spermatozoa nuclei from the CG displayed weak aniline blue staining, while those from the 5-day infected group showed intense staining, indicating chromatin maturation disturbances (Fig. 9C). The infection also reduced mRNA expression of *Crisp1* (Fig. 9D). Ultrastructural analysis of spermatozoa heads in the caudal lumen revealed alterations such as electron-lucent dots within the chromatin, large subacrosomal spaces, and denser accumulation of cellular debris in the lumen in the 5-day post-infection group compared to controls (Fig. 9E).

### Mechanistic crosstalk of inflammatory and cell proliferation pathways

**Figure 10:**
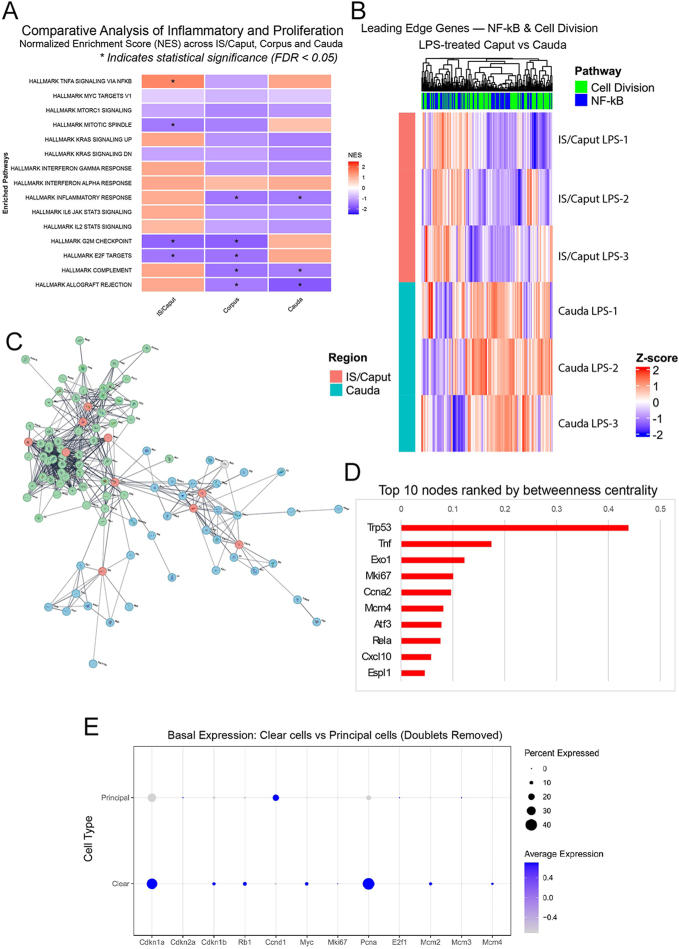
Integrated transcriptomic and interactome analysis reveals inflammation-driven proliferation in epididymal CCs. (A) A heatmap of normalized enrichment scores for selected Hallmark pathways across epididymal regions. Asterisks (*) indicate strict statistical significance (FDR < 0.05). (B) A Z-score expression profile of leading-edge genes driving the NF-kB (blue) and cell division (green) signatures, showing distinct regional clustering. (C) A protein–protein interaction network constructed from leading-edge genes of the co-enriched pathways in the cauda. Nodes are colored by pathway origin (blue: NF-kB; green: cell division). Red nodes denote ‘bridge node’ proteins connecting the two biological networks. In panel D, the top 10 bridge nodes are ranked by betweenness centrality. The presence of key inflammatory (Tnf and Rela) and proliferative (Mki67) regulators highlights the mechanistic crosstalk between the inflammatory stimulus (LPS) and cell cycle entry. (E) A dot plot showing the basal expression of selected cell cycle-related genes in principal *versus* CCs (from uninfected control mice), illustrating the intrinsic transcriptomic priming of CCs.

Reanalysis of our previously published RNA-seq dataset (da Silva *et al.* 2025*a*) revealed that in the cauda, the LPS challenge caused a co-enrichment of pro-inflammatory and cell cycle-related pathways (Fig. 10A). Specifically, we noted a positive enrichment trend for TNF signaling via NF-kB, occurring alongside an upregulation of proliferative signatures, including the G2M checkpoint and E2F targets. Pathways reaching statistical significance (FDR < 0.05) are marked with asterisks.

Furthermore, the expression profile of the leading-edge genes driving both the NF-kB and cell division signatures (Fig. 10B) demonstrated that the transcriptional machinery required for cell proliferation is distinctly upregulated in the cauda CCs alongside the inflammatory cascade. This response contrasts with the more attenuated or differing transcriptional shifts observed in the IS/Caput region.

To elucidate the link between the LPS-induced inflammatory stimulus and proliferative response, we constructed a protein–protein interaction network using the leading-edge genes from the co-enriched NF-kB and cell division pathways (Fig. 10C). To identify the core regulatory molecules orchestrating this crosstalk, we calculated the betweenness centrality for each node.

This topological analysis identified a specific group of central ‘bridge nodes’ that physically connect the inflammatory and proliferative sub-networks (Fig. 10D). Notably, the top bridge proteins included main inflammatory mediators (Tnf, Rela, Cxcl10) alongside regulators of the cell cycle and proliferation (Trp53, Mki67, Ccna2).

Otherwise, a basal-state analysis of the single-cell RNA-seq (Rinaldi *et al.* 2020) showed that PCs barely express the core cell-cycle machinery. Conversely, CCs are in a unique ‘poised state’, consistently co-expressing both the cell cycle ‘brake’ (*Cdkn1a*/*p21*) and the proliferation ‘engine’ (*Mki67*, *Pcna*, *Mcms*) (Fig. 10E). GSEA analysis confirmed the basal enrichment of MYC_TARGETS, E2F_TARGETS, and G2M_CHECKPOINT pathways specifically in CCs (Supplementary Fig. 3).

## Discussion

Studies have demonstrated the impact of SARS-CoV-2 on the male reproductive tract in both humans and animal models (Duarte-Neto *et al.* 2021, Hallak *et al.* 2024, da Silva *et al.* 2025*b*). Several recent clinical and meta-analytic studies have shown that, after COVID-19 infection, men may have lower total sperm count and concentration, reduced progressive and total motility, and increased sperm DNA fragmentation index and, sometimes, worse morphology (Erdik *et al.* 2025). Using the same animal model (k18-hACE2) and infection protocol as in this study, our research team showed that SARS-CoV-2 can infect both testicular (de Oliveira *et al.* 2025) and epididymal (da Silva *et al.* 2025*b*) cells, leading to reduced testosterone levels, increased cytokines, immune cell infiltration, and epithelial damage in both organs. In addition, the epididymal regions respond differently to infection, with the cauda being the most affected (da Silva *et al.* 2025*b*). Therefore, considering the importance of the epididymal cauda to sperm storage (Robaire & Hinton 2015), we focused our analysis on the cauda in this study. Following 5 days of SARS-CoV-2 infection, mediators such as TNF-a, IL-6, and IL-1b, along with CD8^+^ cells and mRNA for *Infa, Infb*, and *Infg*, increased in the cauda. This was followed by basement membrane damage and downregulation of blood–epididymis barrier-related genes. We also observed infected smooth muscle cells, which explain smooth muscle cell death and the reduction in the thickness of the smooth muscle layer. The infection also impaired spermatozoa integrity, specifically affecting the acrosome and chromatin compaction; these findings were corroborated by a decrease in *Crisp1* mRNA expression. CCs expressed hACE2 and were infected by SARS-CoV-2, confirmed by the presence of spike and nucleocapsid protein in these cells. The infection of CCs may be responsible for the increased V-ATPase activity in the apical region and the formation of rows of juxtaposed CCs. Interestingly, the presence of PCNA^+^ and Ki67^+^ CCs in these rows, along with NF-kB immunolabeling, points to a role of NF-kB in the control of proliferation in these cells.

Cytokines play a vital role in both protective and harmful responses during viral infections. Cytokines are key drivers of both immune-mediated virus clearance and immunopathology in various viral infections (Wack *et al.* 2011). In influenza virus infections, interferons (IFN-a/b, IFN-g) and IL-6 have protective functions, while in encephalomyocarditis virus infections, IL-6 and TNF-a serve as protective and exacerbating factors, respectively (Imanishi 2000). In the epididymis, epithelial cells express viral sensors that, when activated, promote the production of type 1 interferons, antiviral proteins, and pro-inflammatory cytokines such as TNF-a and monocyte chemoattractant protein-1 (MCP-1) (Zhu *et al.* 2015). Increased immunolabeling for TNF-a, IL-6, and IL-1b was observed in the cauda stroma 5 days post-infection compared with that in CG. Monocytes and macrophages are especially important for producing pro-inflammatory cytokines that drive innate immune responses and influence T-cell differentiation (Meager & Wadhwa 2013). Furthermore, innate immune cells such as macrophages and epithelial cells release cytokines that initiate inflammation and help direct T-cell subset polarization, thereby contributing to tissue repair (Striz *et al.* 2014). Our previous study showed an increase in mononuclear phagocytes (MPs) in the cauda stroma following SARS-CoV-2 infection (da Silva *et al.* 2025*b*); thus, the elevated cytokines observed here support those previous findings.

The blood–epididymis barrier is essential for male fertility, protecting sperm from the immune system and regulating the composition of the epididymal lumen; however, inflammation can compromise the blood–epididymis barrier, leading to an immune response and reduced sperm function (Gregory & Cyr 2014). In our study, weak claudin immunofluorescence in the epithelium, weak positive basement membrane on silver staining, and downregulation of mRNA for blood–epididymis barrier-related proteins (*Jam-a* and *Ocln*) support the idea that inflammation disturbs the blood–epididymis barrier. Pro-inflammatory cytokines, especially TNF-a, IFN-g, IL-1b, and TGF-b, have been shown to downregulate occludin expression and impair tight junction barriers in various epithelial and endothelial cells. These cytokines decrease occludin mRNA levels, resulting in lower transepithelial resistance and increased paracellular permeability (Van Itallie *et al.* 2010). IFN-g and TNF-a impair epithelial barrier function by internalizing tight junction proteins, including claudin-1 and claudin-4, independently of apoptosis (Bruewer *et al.* 2003). In inflammatory bowel disease and experimental colitis, there is a loss of expression of the epithelial junction adhesion molecule (JAM-A) (Vetrano *et al.* 2008). *Jam-a* downregulation is also observed in CC renal cell carcinoma and is associated with increased cancer cell migration (Gutwein *et al.* 2009). Therefore, the damage to the basement membrane, along with disturbances in the blood–epididymis barrier-related proteins, may result from high cytokine levels, which could impair epithelial cells and sperm.

In addition to cytokines, increased mRNA levels of type I interferons (*Infa* and *Infb*) and type II interferon (Ifng) were observed at 5 days post-infection. Type I and type II interferons (IFNs) play vital roles in the antiviral immune response, with type I IFNs being directly triggered by viral infections and type II IFNs helping to modulate immune response (Lee & Ashkar 2018). The type I IFN system creates an ‘antiviral state’ in cells by activating numerous genes that work to suppress viral replication (Basler & García-Sastre 2002). Both type I and III IFNs are produced rapidly during viral infections, boosting the innate immune response and aiding the switch to adaptive immunity (Levy *et al.* 2011). The dual functions of IFNs, involving both antiviral and immunoregulatory roles, are crucial for balancing effective viral clearance while minimizing tissue damage (Lee & Ashkar 2018).

Type I interferons (IFN-I) act directly on CD8 T cells, promoting their clonal expansion and memory formation in response to viral infection (Kolumam *et al.* 2005). IFN-I receptor signaling in CD8 T cells is essential to produce effector and memory cells. Similarly, interferon-g (IFN-g) directly enhances CD8 T cell development during acute viral infections (Whitmire *et al.* 2005). In this study, in addition to the INFs upregulation, we observed increased CD8 immunolabeling in the stroma of the cauda at 5 days post-infection compared to CG, confirming infiltration of these cells. CD8^+^ T cells play a vital role in immune defense against viral infections and cancers. These cytotoxic T lymphocytes recognize infected cells through MHC-I-dependent processes and eliminate them via mechanisms such as the release of perforin and granzymes, death receptor engagement, and the secretion of antiviral factors (Gulzar & Copeland 2004). These cells are primarily located in the subepithelial layer and interstitial tissues of the epididymis (Güney Saruhan *et al.* 2018). The concomitant increase in cytokines, IFN upregulation, and recruitment of CD8+ cells indicates a robust epididymal immune response to viral infection, promoting the clearance of infected cells.

Smooth muscle cells in the epididymal duct are vital for proper sperm transit (Elfgen *et al.* 2018). In this study, smooth muscle cells were infected with SARS-CoV-2, as indicated by colocalization of spike or nucleocapsid with actin. The infection led to atrophy of the muscular layer due to SMC death, as confirmed by ultrastructural analysis. While earlier studies suggested that muscle pathology during COVID-19 might be predominantly immune-mediated rather than directly caused by infection, recent advances have challenged this paradigm in smooth muscle. Indeed, Richards *et al.* (2024) recently demonstrated that smooth muscle cells are susceptible to SARS-CoV-2 infection, and our *in situ* findings corroborate this smooth muscle tropism. These smooth muscle cells are crucial for effective sperm transport, supporting sperm maturation and male fertility (Elfgen *et al.* 2018). They are regulated by various factors, including products from mast cells and macrophages, and can produce extracellular matrix components and other secreted factors (Mayerhofer 2013). Disruptions in smooth muscle cell function or structure may impair sperm maturation and cause male infertility, underscoring the importance of smooth muscle cells in reproductive health (Mayerhofer 2013, Elfgen *et al.* 2018). Therefore, muscular layer atrophy after infection could disrupt sperm maturation. It is also noteworthy that smooth muscle cell infection may be one route by which SARS-CoV-2 reaches the epithelium, since viral particles in the blood initially bind to smooth muscle cells before infecting epididymal epithelial cells.

Five days after infection, we observed *Crisp1* downregulation and spermatozoa alterations, including decreased PNA labeling (an acrosomal marker), chromatin immaturity (as indicated by aniline blue staining), and ultrastructural alterations in the spermatozoa nucleus. CRISP1 (cysteine-rich secretory protein 1) is an epididymal protein that binds to sperm during maturation, helping the regulation of key processes for fertility (Roberts *et al.* 2006). CRISP1 is secreted by the epididymal epithelium and binds to maturing spermatozoa, inhibiting premature capacitation by blocking signaling cascades, including those involving calcium channels, thereby preventing hyperactivation until spermatozoa reach the female tract (Ernesto *et al.* 2015). CRISP1 knockdown disrupts sperm DNA integrity and epididymal function, revealing its essential role in fertility beyond gamete fusion (Sulzyk *et al.* 2025). The presence of cellular debris in the lumen alongside high cytokine levels indicates an oxidative environment. Oxidative stress significantly contributes to sperm damage and male infertility by disrupting the balance between reactive oxygen species (ROS) production and antioxidant defenses in semen. Spermatozoa’s polyunsaturated fatty acids are highly susceptible to oxidative attack by excessive ROS, leading to lipid peroxidation and membrane disruption, reducing motility and impairing fertilization capacity (Wang *et al.* 2025). Thus, epithelial and smooth muscle layer damage, coupled with *Crisp1* downregulation and increased cytokine levels, may contribute to the impaired sperm maturation process observed 5 days post-SARS-CoV-2 infection.

Proton-secreting CCs help maintain an adequate luminal pH, which is critical for sperm maturation and quiescence (Breton & Brown 2013). This process is carried out by the V-ATPase proton pump in the apical membrane of the CCs (Breton & Brown 2013). Data from CC RNA-seq show that these cells express the enzyme ACE2 (Battistone *et al.* 2019*a*,*b*). In our study, we also found that CCs from our transgenic mice expressed hACE2, allowing these cells to be infected by SARS-CoV-2, as confirmed by the colocalization of V-ATPase with spike or nucleocapsid proteins. Infection over five days resulted in increased V-ATPase immunolabeling in the apical region of CCs, suggesting intense recruitment of this proton pump to the apical surface. At five days post-infection, *Foxi1* mRNA levels increased. *Foxi1* is a transcription factor that positively regulates V-ATPase expression and activity, especially in specialized epithelial cells, including epididymal CCs (Vidarsson *et al.* 2009). Therefore, the upregulation of *Foxi1* observed here may serve as a compensatory mechanism to preserve the acidic environment of the epididymal lumen. However, we cannot rule out the possibility that *Foxi1* upregulation is related to CC proliferation.

V-ATPase activity is also regulated by various mechanisms, including nitric oxide (NO), angiotensin II (Ang II) signaling via basal cells, and ATP/adenosine-mediated activation (Shum *et al.* 2011, Battistone *et al.* 2019*a*,*b*). Studies have shown that COVID-19 patients, especially those with critical illness, exhibit elevated levels of Ang II in their plasma. Camargo *et al.* (2022) reported significantly higher Ang II levels in critically ill patients compared to those with severe COVID-19. Similarly, Wu *et al.* (2020) found that 90.2% of COVID-19 cases had higher Ang II levels, with 100% of critically ill patients showing elevated levels (Liu *et al.* 2021). In our previous study, we also demonstrated that SARS-CoV-2 infection increased inducible nitric oxide synthase (iNOS) in the cauda epididymis (da Silva *et al.* 2025*b*). Furthermore, we have recently shown elevated Ang II levels in the testes of this same model at 5 days post-infection (de Oliveira *et al.* 2026). Therefore, the translocation of V-ATPase to the CC apical membrane, observed in this study, may be linked to Ang II and NO, which could negatively affect sperm cell maturation and storage.

Besides their role in maintaining luminal pH balance (Breton & Brown 2013), CCs also participate in the immune response of the epididymis, expressing TNF-a during SARS-CoV-2 infection (da Silva *et al.* 2025*b*) and showing a pro-inflammatory transcriptomic shift upon LPS stimulation (Battistone *et al.* 2019*a*,*b*, da Silva *et al.* 2025*a*). Interestingly, in our SARS-CoV-2 model, we observed, for the first time, juxtaposed CC rows containing numerous PCNA-positive and Ki67-positive cells, indicating active proliferation, an uncommon event in terminally differentiated epithelial cells. In the kidney, the proton-secreting intercalated cells can expand in response to increased acid load or systemic acidosis, reflecting an adaptive remodeling of the epithelium (Cheval *et al.* 2021). Although proliferation increased at both 2 and 5 days post-infection, we observed a decrease in PCNA + CCs at 5 days post-infection compared to 2 days. This finding may suggest an exhaustion of the proliferative ‘wave’ at this period; however, further studies are necessary to establish the complete mechanisms governing CC proliferation within the epididymis.

We also observed that the PCNA-positive cells were also positive for the phosphorylated NF-kB, suggesting that proliferation might be part of the CC immune response. To determine whether this proliferation was virus-specific or a general response to inflammation, we reanalyzed the RNA-seq dataset from CCs stimulated with LPS for only 1 h (da Silva *et al.* 2025*a*), confirming that CCs rapidly activate proliferative programs in parallel with inflammatory pathways upon stimulation. These findings align with our *in vivo* SARS-CoV-2 data, which show upregulation of interferon mRNAs and increased TNF-a immunostaining (da Silva *et al.* 2025*b*). Notably, the regional analysis revealed a distinct activation signature (E2F/G2M pathway) specifically in the cauda epididymis.

To clarify the mechanistic basis of this inflammatory-proliferative link, we constructed the PPI network using key genes from the co-activated NF-kB and cell division pathways. PPI networks revealed distinct inflammatory and proliferative modules connected by essential ‘bridge nodes’. The presence of the NF-kB subunit Rela among the top 10 shared bridges, alongside TNF and Cxcl10, as well as proteins related to the cell cycle and division such as Mki67, Ccna2, and Trp53, provides strong evidence for its central role in mediating this crosstalk between the two pathways. This *in silico* prediction of a direct link between NF-kB activation and proliferation was experimentally validated in our primary SARS-CoV-2 infection model. Using immunofluorescence, we directly demonstrated the colocalization of phosphorylated (active) NF-kB-p and the proliferation marker PCNA in the same CCs at 2 days post-infection.

However, this raised two important questions: i) how could the proliferation program be quickly activated (in just 1 h) in the LPS model? ii) Why do only CCs proliferate, given that we also observed NF-kB-p activation in principal cells? To answer this, we examined the basal state of a public scRNA-seq atlas of the normal mouse epididymis (Rinaldi *et al.* 2020) using a pseudo-bulk DEA approach. A basal-state comparison revealed that principal cells barely express the core cell-cycle machinery. Conversely, CCs are in a unique ‘poised state’, consistently co-expressing both the cell cycle ‘brake’ (*Cdkn1a*/*p21*) and the proliferation ‘engine’ (*Mki67*, *Pcna*, *Mcms*). GSEA confirmed the basal enrichment of MYC_TARGETS, E2F_TARGETS, and G2M_CHECKPOINT pathways specifically in CCs. This poised state provides a clear mechanistic explanation: CCs respond rapidly because their machinery is already assembled, requiring only the inflammatory signal (NF-kB/IFN → bridge genes) to override the basal Cdkn1a brake; PCs cannot proliferate because they lack the engine altogether. These combined findings, from the *in vivo* SARS-CoV-2 model, the *in silico* LPS model, and the *in silico* scRNA-seq analysis, strongly support our main hypothesis: proliferation is a fundamental, programmed response of CCs to immune disturbances, enabled by their unique poised state and linked through direct molecular crosstalk from inflammatory pathways such as NF-kB and interferon signaling. Considering that CCs exist in this poised state and that, upon stimulation, these cells proliferate, we elaborated a scheme to illustrate a side view of the CCs rows’ disposition along the epididymal epithelium layer, forming ‘islands’ of CCs with a mitotic potential, which is activated by stimulus, such as the viral infection. Further studies are necessary to clarify the disposition and function of these CCs in the epididymal epithelium. Importantly, the reanalysis of RNA-seq and scRNA-seq datasets did not aim to directly compare the specific mechanisms of SARS-CoV-2 at 5 days post-infection with the acute effects of a 1 h LPS model. Instead, these datasets were utilized to establish a conceptual framework linking acute inflammatory stimuli to cell division. This approach helps to explain the rapid, previously unreported proliferative response observed in CCs. While our network analysis identifies key molecular bridges, further studies are required to elucidate the causative link between the inflammatory cascade and CC proliferation.

### Study limitations

We recognize important limitations of the *in vivo* model used in this study. The K18-hACE2 mouse is a highly susceptible model that quickly develops severe, lethal disease marked by systemic hyperinflammation. In human COVID-19, systemic cytokine-driven pathology, fever, and endocrine issues are well known to impact reproductive tissues. Since we could not measure core body temperature or circulating reproductive hormones (e.g. testosterone, LH, FSH) over time during the acute BSL-3 infection protocols, we cannot definitively separate the direct effects of SARS-CoV-2 replication from the effects of severe systemic illness. Therefore, the tissue changes described here likely result from a combination of local viral presence and the broader systemic inflammatory response. Future studies using non-lethal, mild-to-moderate infection models, such as Syrian hamsters or mouse-adapted viral strains, are needed to isolate these factors and determine how generalizable these findings are to typical human disease.

## Conclusion

This study characterizes the severe immunopathology caused by SARS-CoV-2 in the cauda epididymis. We show that the viral infection triggers a strong local inflammatory response, marked by increased cytokine and interferon levels, infiltration of CD8^+^ T cells, and subsequent downregulation of genes related to the blood–epididymis barrier. In addition, we identify two new cellular mechanisms of virus-induced damage: i) infection and apoptotic death of smooth muscle cells, resulting in muscular layer atrophy, and ii) activation of a previously unrecognized proliferative program in CCs. This proliferation is not a virus-specific artifact but a fundamental response to immune stimuli. We provide a comprehensive mechanistic explanation, demonstrating i) pathway crosstalk from NF-kB to the cell cycle machinery via key bridge nodes in an LPS model, ii) validation *in vivo*, and iii) the ‘poised state’ of CCs, a biological prerequisite that allows their rapid proliferation under inflammatory stimuli. These findings establish CCs as key mediators of the epididymal immune response, initiating synchronized inflammatory and proliferative programs. Along with muscular damage, these results highlight how acute viral infections compromise the microenvironment necessary for sperm maturation and storage, positioning CCs as immune sentinels in viral epididymitis.

## Supplementary materials





## Declaration of interest

The authors declare that there is no conflict of interest that could be perceived as prejudicing the impartiality of the work reported.

## Funding

This work was supported by the Fundação de Amparo à Pesquisa do Estado de São Paulo (2021/07207-6; 2022/10560-2), Tertiary Education Trust Fund; Nigeria (Grant number: TETFUND/ES/TSAS/MOU/FARA/2024/VOL.I) and Coordenação de Aperfeiçoamento de Pessoal de Nível Superior – CAPES (Cod. 001); CNPq (309301/2021-1 and 310256/2022-4).

## Author contribution statement

AASS and ES-C were involved in the study design and conceptualization. AASS and OSA performed experiments and data analysis. AASS, OSA, MAB, PSC, and ES-C were involved in data interpretation. AASS and ES-C wrote the original manuscript. All authors contributed to the writing of the manuscript, made critical comments, and approved the final version.

## References

[bib1] Ardestani Zadeh A & Arab D 2021 COVID-19 and male reproductive system: pathogenic features and possible mechanisms. J Mol Histol 52 869–878. (10.1007/s10735-021-10003-3) 34232425 PMC8260577

[bib2] Basler CF & García-Sastre A 2002 Viruses and the type I interferon antiviral system: induction and evasion. Int Rev Immunol 21 305–337. (10.1080/08830180213277) 12486817

[bib3] Battistone MA, Merkulova M, Park YJ, et al. 2019a Unravelling purinergic regulation in the epididymis: activation of V-ATPase-dependent acidification by luminal ATP and adenosine. J Physiol 597 1957–1973. (10.1113/jp277565) 30746715 PMC6441927

[bib4] Battistone MA, Spallanzani RG, Mendelsohn AC, et al. 2019b Novel role of proton-secreting epithelial cells in sperm maturation and mucosal immunity. J Cell Sci 133 jcs233239. (10.1242/jcs.233239) 31636115 PMC7003979

[bib5] Breton S & Brown D 2013 Regulation of luminal acidification by the V-ATPase. Physiology 28 318–329. (10.1152/physiol.00007.2013) 23997191 PMC3768094

[bib6] Bruewer M, Luegering A, Kucharzik T, et al. 2003 Proinflammatory cytokines disrupt epithelial barrier function by apoptosis-independent mechanisms. J Immunol 171 6164–6172. (10.4049/jimmunol.171.11.6164) 14634132

[bib7] Camargo RL, Bombassaro B, Monfort-Pires M, et al. 2022 Plasma angiotensin II is increased in critical coronavirus disease 2019. Front Cardiovasc Med 9 847809. (10.3389/fcvm.2022.847809) 35811697 PMC9263116

[bib8] Cerri PS & Sasso-Cerri E 2003 Staining methods applied to glycol methacrylate embedded tissue sections. Micron 34 365–372. (10.1016/s0968-4328(03)00098-2) 14680922

[bib9] Cheval L, Viollet B, Klein C, et al. 2021 Acidosis-induced activation of distal nephron principal cells triggers Gdf15 secretion and adaptive proliferation of intercalated cells. Acta Physiol 232 e13661. (10.1111/apha.13661) 33840159

[bib10] Cox MA, Kahan SM & Zajac AJ 2013 Anti-viral CD8 T cells and the cytokines that they love. Virology 435 157–169. (10.1016/j.virol.2012.09.012) 23217625 PMC3580945

[bib11] da Silva AAS, de Santi F, Hinton BT, et al. 2023 Venlafaxine increases aromatization, reduces apical V-ATPase in clear cells and induces increased number of mast cells and smooth muscle cells death in rat cauda epididymis. Life Sci 315 121329. (10.1016/j.lfs.2022.121329) 36584913

[bib12] da Silva AAS, Barrachina F, Avenatti MC, et al. 2025a Proton-secreting cells as drivers of inflammation and sperm dysfunction in LPS-induced epididymitis. Function 6 zqaf023. (10.1093/function/zqaf023) 40455583 PMC12203219

[bib13] da Silva AAS, de Oliveira SA, Battistone MA, et al. 2025b hACE2 upregulation and participation of macrophages and clear cells in the immune response of epididymis to SARS-CoV-2 in K18-hACE2 mice. Andrology 13 1509–1529. (10.1111/andr.13755) 39363435

[bib14] de Oliveira SA, da Silva AAS, Hinton BT, et al. 2025 SARS-CoV-2 exploits steroidogenic machinery, triggers lipid metabolism for viral replication and induces immune response in Leydig cells of K18-hACE2 mice. Front Cell Infect Microbiol 15 1538461. (10.3389/fcimb.2025.1538461) 40496014 PMC12149123

[bib15] de Oliveira SA, da Silva AAS, Hinton BT, et al. 2026 Ultrastructural features, immune response, and junctional proteins in the seminiferous epithelium of SARS-CoV-2-infected mice. Int J Mol Sci 27 691. (10.3390/ijms27020691) 41596343 PMC12840741

[bib16] Duarte-Neto AN, Caldini EG, Gomes-Gouvêa MS, et al. 2021 An autopsy study of the spectrum of severe COVID-19 in children: from SARS to different phenotypes of MIS-C. EClinicalMedicine 35 100850. (10.1016/j.eclinm.2021.100850) 33937731 PMC8072136

[bib17] Elfgen V, Mietens A, Mewe M, et al. 2018 Contractility of the epididymal duct: function, regulation and potential drug effects. Reproduction 156 R125–R141. (10.1530/rep-17-0754) 30304934

[bib18] Elizagaray ML, Barrachina F, Avenatti MC, et al. 2025 Chronic inflammation drives epididymal tertiary lymphoid structure formation and autoimmune fertility disorders in mice. Nat Commun 16 8742. (10.1038/s41467-025-63514-y) 41034192 PMC12488923

[bib19] Erdik A, Gokce AM & Gokce A 2025 The effects of COVID-19 on semen parameter values in healthy males: a single-centre, retrospective study. PeerJ 13 e19864. (10.7717/peerj.19864) 40900756 PMC12401025

[bib69] Ernesto JI, Weigel Muñoz M, Battistone MA, et al. 2015 CRISP1 as a novel CatSper regulator that modulates sperm motility and orientation during fertilization. J Cell Biol 210 1213–1224. (10.1083/jcb.201412041) 26416967 PMC4586743

[bib20] Gomori G 1937 Silver impregnation of reticulum in paraffin sections. Am J Path 13 993–1002. 19970363 PMC1911151

[bib22] Gregory M & Cyr DG 2014 The blood-epididymis barrier and inflammation. Spermatogenesis 4 e979619. (10.4161/21565562.2014.979619) 26413391 PMC4581054

[bib65] Gu Z, Eils R & Schlesner M 2016 Complex heatmaps reveal patterns and correlations in multidimensional genomic data. Bioinformatics 32 2847–2849. (10.1093/bioinformatics/btw313) 27207943

[bib23] Gulzar N & Copeland KF 2004 CD8+ T-cells: function and response to HIV infection. Curr HIV Res 2 23–37. (10.2174/1570162043485077) 15053338

[bib24] Güney Saruhan B, Sağsöz H, Akbalık E, et al. 2018 Distribution of CD68-CD8-MHCI- and MHCII-positive cells in the bull and ram testis and epididymis. Anat Histol Embryol 47 313–321. (10.1111/ahe.12354) 29527795

[bib25] Gutwein P, Schramme A, Voss B, et al. 2009 Downregulation of junctional adhesion molecule-A is involved in the progression of clear cell renal cell carcinoma. Biochem Biophys Res Commun 380 387–391. (10.1016/j.bbrc.2009.01.100) 19250634

[bib26] Hallak J, Caldini EG, Teixeira TA, et al. 2024 Transmission electron microscopy reveals the presence of SARS-CoV-2 in human spermatozoa associated with an ETosis-like response. Andrology 12 1799–1807. (10.1111/andr.13612) 38469742

[bib27] He Y, Wang J, Ren J, et al. 2021 Effect of COVID-19 on Male reproductive system – a systematic review. Front Endocrinol 12 677701. (10.3389/fendo.2021.677701) PMC8190708 34122351

[bib28] Hoffmann K & Gopal M 2024 Paediatric acute epididymo-orchitis temporally related to SARS-CoV-2 infection: a case series and review of the literature. J Pediatr Urol 20 91–94. (10.1016/j.jpurol.2023.09.017) 37806834

[bib67] Imanishi J 2000 Expression of cytokines in bacterial and viral infections and their biochemical aspects. J Biochem 127 525–530. (10.1093/oxfordjournals.jbchem.a022636) 10739941

[bib29] Inouye D, Baker Z, Peña A, et al. 2023 Epididymitis, orchitis, and epididymo-orchitis associated with SARS-CoV-2 infection in pediatric patients: a systematic review. Front Urol 2 1092192. (10.3389/fruro.2022.1092192)

[bib30] Johnston DS, Jelinsky SA, Bang HJ, et al. 2005 The mouse epididymal transcriptome: transcriptional profiling of segmental gene expression in the epididymis. Biol Reprod 73 404–413. (10.1095/biolreprod.105.039719) 15878890

[bib31] Kolumam GA, Thomas S, Thompson LJ, et al. 2005 Type I interferons act directly on CD8 T cells to allow clonal expansion and memory formation in response to viral infection. J Exp Med 202 637–650. (10.1084/jem.20050821) 16129706 PMC2212878

[bib64] Law CW, Chen Y, Shi W, et al. 2014 Voom: Precision weights unlock linear model analysis tools for RNA-seq read counts. Genome Biol 15 R29. (10.1186/gb-2014-15-2-r29) 24485249 PMC4053721

[bib32] Lee AJ & Ashkar AA 2018 The dual nature of type I and type II interferons. Front Immunol 9 2061. (10.3389/fimmu.2018.02061) 30254639 PMC6141705

[bib33] Levy DE, Marié IJ & Durbin JE 2011 Induction and function of type I and III interferon in response to viral infection. Curr Opin Virol 1 476–486. (10.1016/j.coviro.2011.11.001) 22323926 PMC3272644

[bib34] Liu N, Hong Y, Chen R, et al. 2021 High rate of increased level of plasma angiotensin II and its gender difference in Covid-19: an analysis of 55 hospitalized patients with Covid-19 in a single Hospital, Wuhan, China. J Clin Toxicol S16 1.

[bib35] Ma W, Li S, Ma S, et al. 2016 Zika virus causes testis damage and leads to Male infertility in mice. Cell 167 1511–1524.e10. (10.1016/j.cell.2016.11.016) 27884405

[bib36] Mayerhofer A 2013 Human testicular peritubular cells: more than meets the eye. Reproduction 145 R107–R116. (10.1530/rep-12-0497) 23431272

[bib37] McCray PB Jr, Pewe L, Wohlford-Lenane C, et al. 2007 Lethal infection of K18-hACE2 mice infected with severe acute respiratory syndrome coronavirus. J Virol 81 813–821. (10.1128/jvi.02012-06) 17079315 PMC1797474

[bib38] Meager A & Wadhwa M 2013 An overview of cytokine regulation of inflammation and immunity. In Encyclopedia of Life Sciences, edn 1. Wiley. (10.1002/9780470015902.a0024658)

[bib39] Michel V, Duan Y, Stoschek E, et al. 2016 Uropathogenic Escherichia coli causes fibrotic remodelling of the epididymis. J Pathol 240 15–24. (10.1002/path.4748) 27218225

[bib40] Mital P, Hinton BT & Dufour JM 2011 The blood-testis and blood-epididymis barriers are more than just their tight junctions. Biol Reprod 84 851–858. (10.1095/biolreprod.110.087452) 21209417 PMC4574632

[bib41] Richards A, Khalil AS, Friesen M, et al. 2024 SARS-CoV-2 infection of human pluripotent stem cell-derived vascular cells reveals smooth muscle cells as key mediators of vascular pathology during infection. Nat Commun 15 10754. (10.1038/s41467-024-54917-4) 39737992 PMC11685814

[bib42] Rinaldi VD, Donnard E, Gellatly K, et al. 2020 An atlas of cell types in the mouse epididymis and vas deferens. Elife 9 e55474. (10.7554/elife.55474) 32729827 PMC7426093

[bib66] Robaire B & Hinton BT 2015 The epididymis. In Knobil and Neill’s Physiology of Reproduction: Two-Volume Set, 4th ed. Eds TM Plant & AJ Zeleznik. Elsevier. (10.1016/B978-0-12-397175-3)

[bib43] Roberts KP, Ensrud KM, Wooters JL, et al. 2006 Epididymal secreted protein Crisp-1 and sperm function. Mol Cell Endocrinol 250 122–127. (10.1016/j.mce.2005.12.034) 16414181

[bib62] Sasso-Cerri E & Cerri PS 2008 Morphological evidences indicate that the interference of cimetidine on the peritubular components is responsible for detachment and apoptosis of Sertoli cells. Reprod Biol Endocrinol 6 18. (10.1186/1477-7827-6-18) 18471284 PMC2413234

[bib44] Schmidt ME & Varga SM 2018 The CD8 T cell response to respiratory virus infections. Front Immunol 9 678. (10.3389/fimmu.2018.00678) 29686673 PMC5900024

[bib45] Sheikhzadeh Hesari F, Hosseinzadeh SS & Asl Monadi Sardroud MA 2021 Review of COVID-19 and male genital tract. Andrologia 53 e13914. (10.1111/and.13914) 33236375 PMC7744899

[bib46] Sheng Z, Gao N, Fan D, et al. 2021 Zika virus disrupts the barrier structure and absorption/secretion functions of the epididymis in mice. PLoS Negl Trop Dis 15 e0009211. (10.1371/journal.pntd.0009211) 33667230 PMC7968736

[bib47] Shum WW, Ruan YC, Da Silva N, et al. 2011 Establishment of cell-cell cross talk in the epididymis: control of luminal acidification. J Androl 32 576–586. (10.2164/jandrol.111.012971) 21441423 PMC3753098

[bib48] Situ J, Wang W, Long F, et al. 2020 Hepatitis E viral infection causes testicular damage in mice. Virology 541 150–159. (10.1016/j.virol.2019.12.009) 32056713

[bib68] Striz I, Brabcova E, Kolesar L, et al. 2014 Cytokine networking of innate immunity cells: a potential target of therapy. Clin Sci 126 593–612. (10.1042/CS20130497) 24450743

[bib49] Sulzyk V, Curci L, González LN, et al. 2025 The epididymis contributes to sperm DNA integrity and early embryo development through cysteine-rich secretory proteins. Elife 13 RP97105. (10.7554/elife.97105.3) 40293787 PMC12037180

[bib50] Terquem A & Dadoune JP 1983 Aniline blue staining of human spermatozoon chromatin. Evaluation of nuclear maturation. In The Sperm Cell, pp 249–252. Ed J AndrC. The Hague: MartinusNijhoff Publishers.

[bib51] Turner TT, Mammen T, Kavoussi P, et al. 2011 Cytokine responses to E. Coli-induced epididymitis in the rat: blockade by vasectomy. Urology 77 1507.e9–1507.e14. (10.1016/j.urology.2011.02.037) 21529899

[bib63] Untergasser A, Cutcutache I, Koressaar T, et al. 2012 Primer3--new capabilities and interfaces. Nucleic Acids Res 40 e115. (10.1093/nar/gks596) 22730293 PMC3424584

[bib52] Van Itallie CM, Fanning AS, Holmes J, et al. 2010 Occludin is required for cytokine-induced regulation of tight junction barriers. J Cell Sci 123 2844–2852. (10.1242/jcs.065581) 20663912 PMC2915885

[bib53] Vetrano S, Rescigno M, Rosaria Cera M, et al. 2008 Unique role of junctional adhesion molecule-a in maintaining mucosal homeostasis in inflammatory bowel disease. Gastroenterology 135 173–184. (10.1053/j.gastro.2008.04.002) 18514073

[bib54] Vidarsson H, Westergren R, Heglind M, et al. 2009 The forkhead transcription factor Foxi1 is a master regulator of vacuolar H-ATPase proton pump subunits in the inner ear, kidney and epididymis. PLoS One 4 e4471. (10.1371/journal.pone.0004471) 19214237 PMC2637605

[bib55] Wack A, Openshaw P & O’Garra A 2011 Contribution of cytokines to pathology and protection in virus infection. Curr Opin Virol 1 184–195. (10.1016/j.coviro.2011.05.015) 22440716

[bib56] Wang Y, Fu X & Li H 2025 Mechanisms of oxidative stress-induced sperm dysfunction. Front Endocrinol 16 1520835. (10.3389/fendo.2025.1520835) PMC11835670 39974821

[bib57] Wherry EJ & Ahmed R 2004 Memory CD8 T-cell differentiation during viral infection. J Virol 78 5535–5545. (10.1128/jvi.78.11.5535-5545.2004) 15140950 PMC415833

[bib58] Whitmire JK, Tan JT & Whitton JL 2005 Interferon-gamma acts directly on CD8+ T cells to increase their abundance during virus infection. J Exp Med 201 1053–1059. (10.1084/jem.20041463) 15809350 PMC2213135

[bib59] Wu Z, Hu R, Zhang C, et al. 2020 Elevation of plasma angiotensin II level is a potential pathogenesis for the critically ill COVID-19 patients. Crit Care 24 290. (10.1186/s13054-020-03015-0) 32503680 PMC7273814

[bib60] Zhu W, Zhao S, Liu Z, et al. 2015 Pattern recognition receptor-initiated innate antiviral responses in mouse epididymal epithelial cells. J Immunol 194 4825–4835. (10.4049/jimmunol.1402706) 25840915

