# Supplementary figures and images for: SARS-CoV-2 triggers an NF-kB-driven proliferative response in epididymal clear cells of K18-hACE2 mice
Source: Reprod Fertil. 2026 May 28;7(2):RAF250197. doi: 10.1530/RAF-25-0197 (PMC13232600; doi:10.1530/RAF-25-0197)

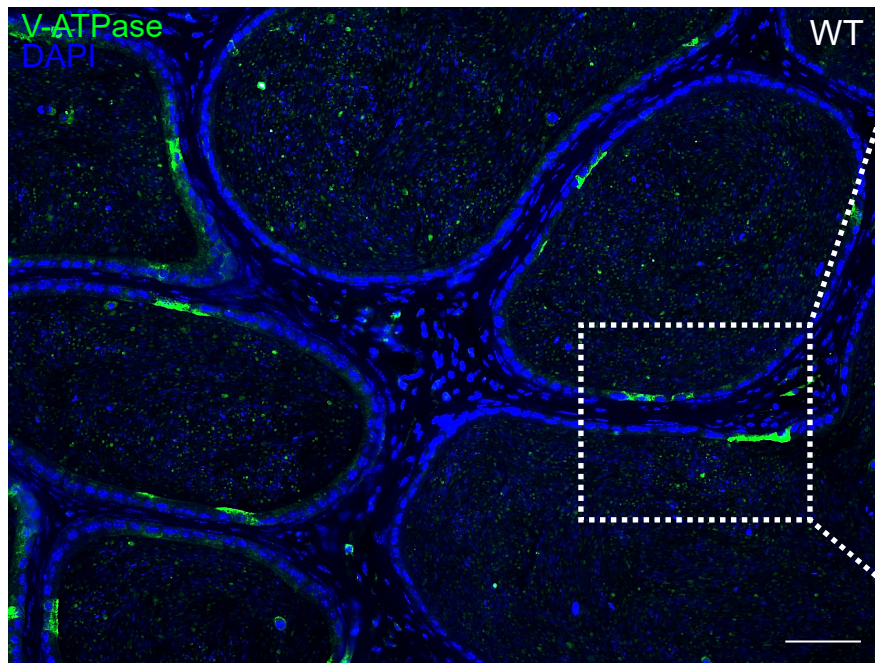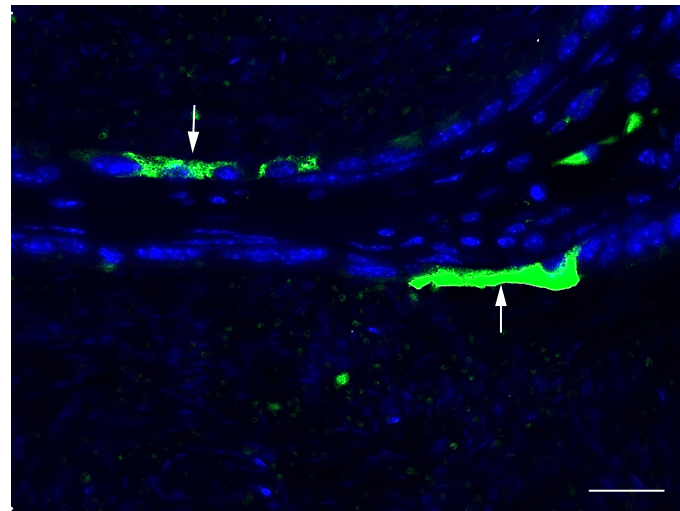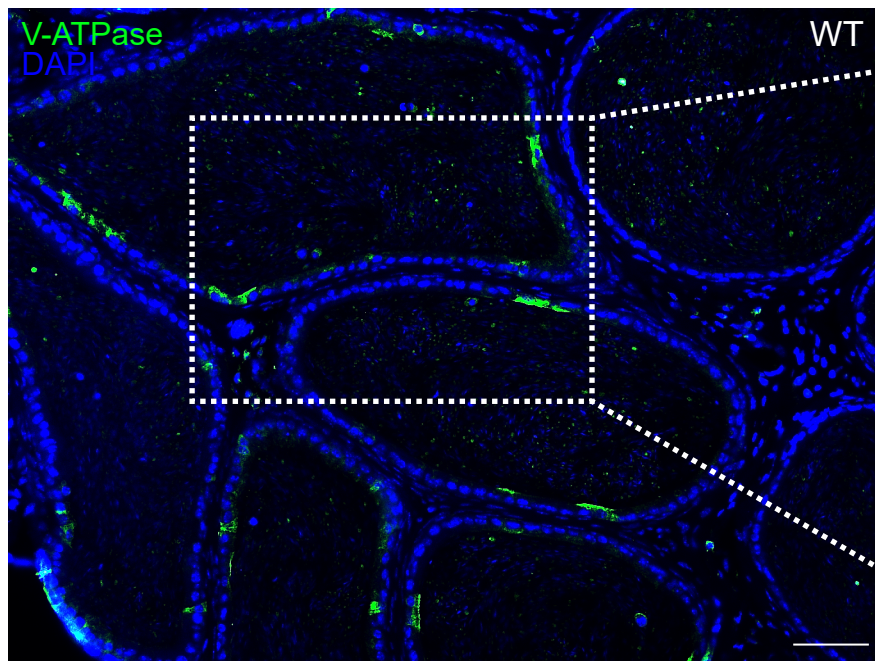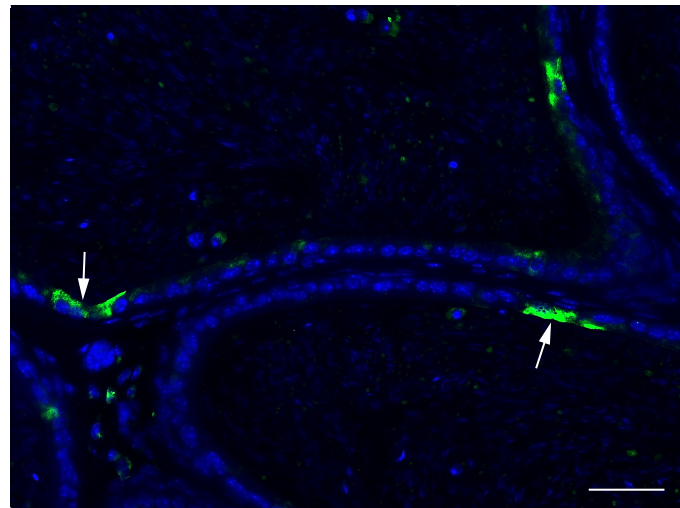

A

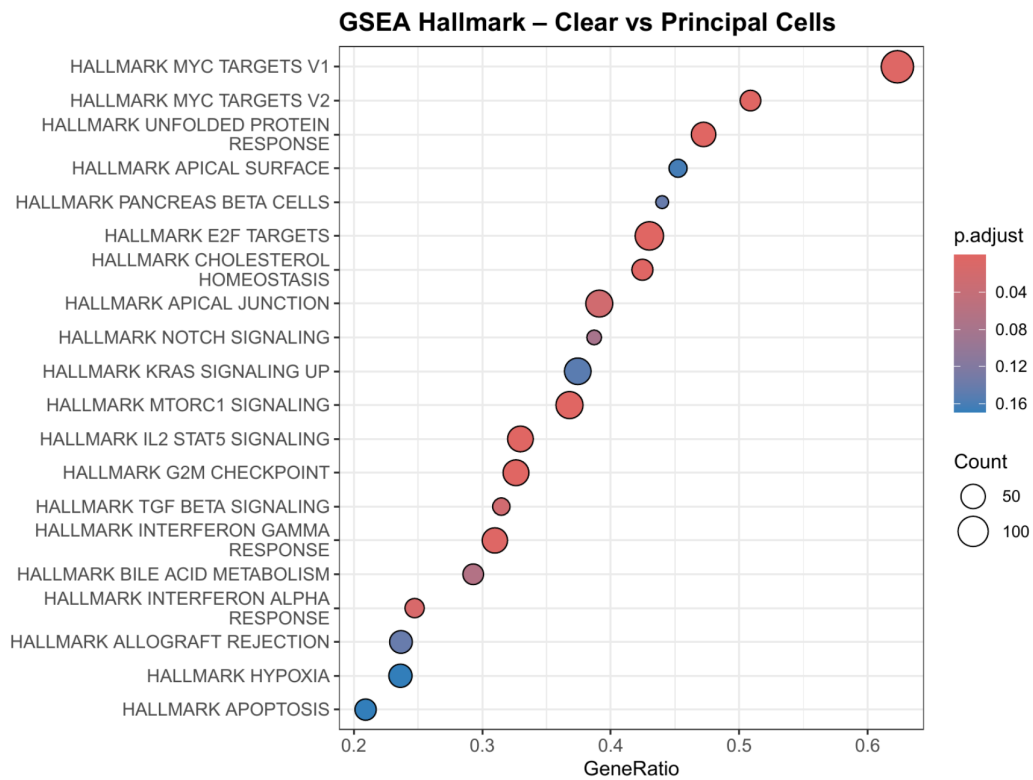

B

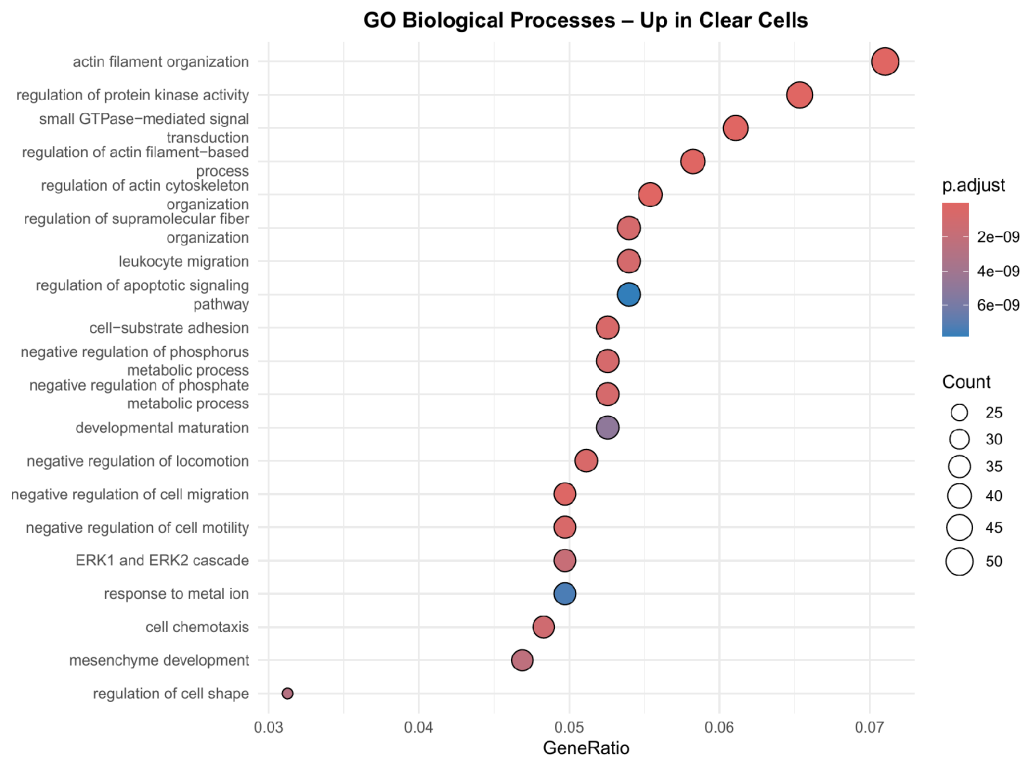

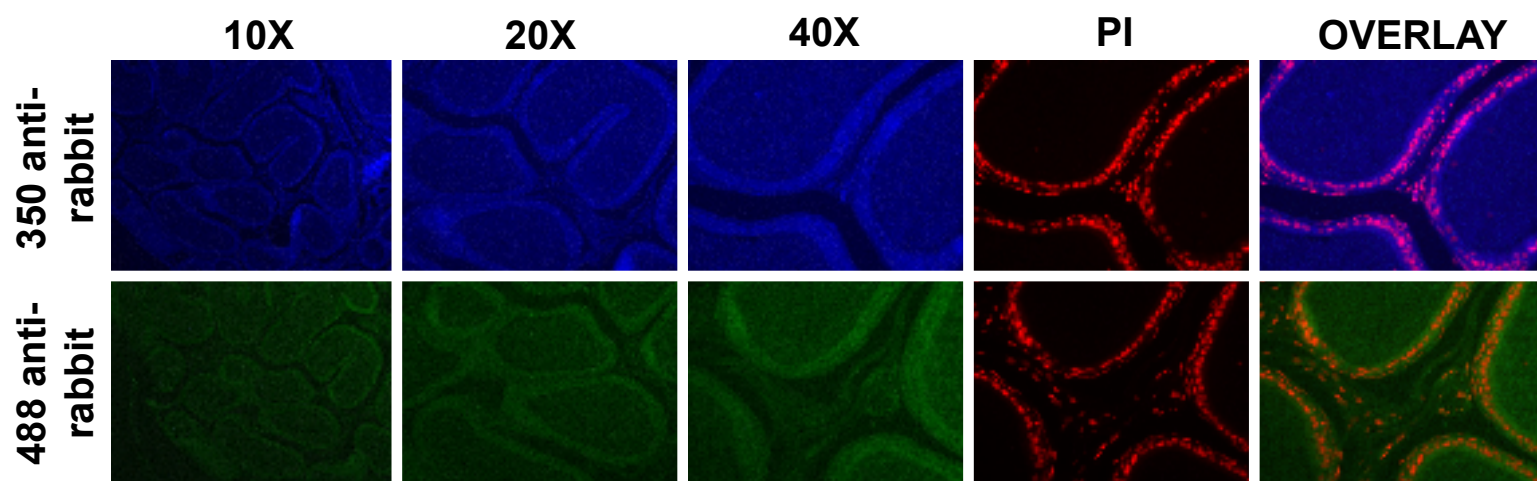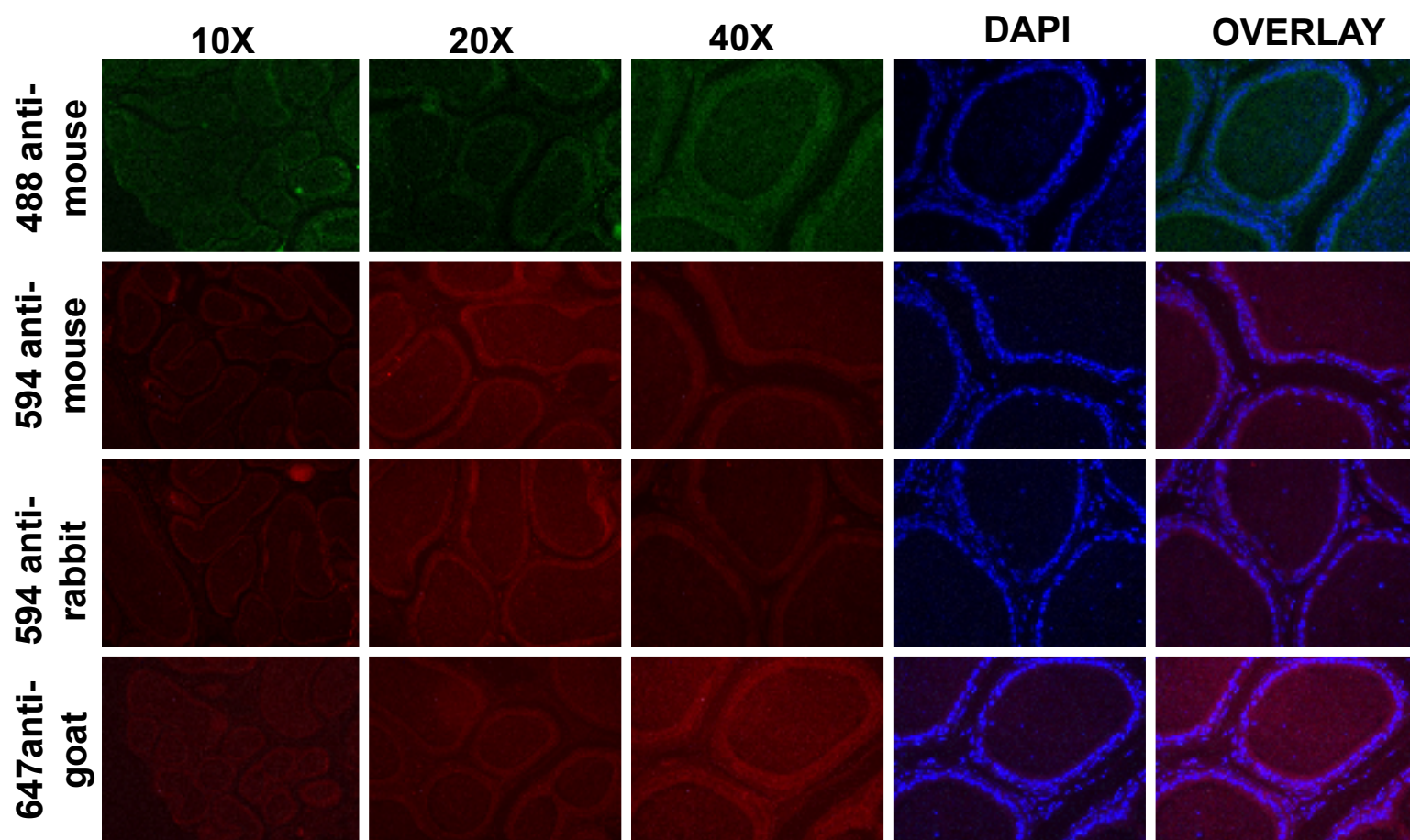

Supplement: Supplementary file 1 [file supplementary_figures.pdf]
