# Supplementary material for: SARS-CoV-2 triggers an NF-kB-driven proliferative response in epididymal clear cells of K18-hACE2 mice
Source: Reprod Fertil. 2026 May 28;7(2):RAF250197. doi: 10.1530/RAF-25-0197 (PMC13232600; doi:10.1530/RAF-25-0197)
Supplement: Supplementary file 2 [file supplementary_table.pdf]

| Pathway | p-value | (FDR) | Log2 Error | Enrichment score | Normalized Enrichment Score (NES) | Gene set size | Leading Edge Genes |
|---|---|---|---|---|---|---|---|
| HALLMARK_TGF_BETA_SIGNALING | 0.003 | 0.043 | 0.432 | 0.552 | 1.69 | 52 | Bmp2/Thbs1/Nog/Klf10/Smad6/Id2/Skil/Ppp1r15a/Smurf2/Wwtr1/Slc20a1/Trim33/Id1/Sptbn1/Tgif1/Tgfbr1/Cdh1/Ifngr2/Bmpr1a/Smad3/Smad1 |
| HALLMARK_TNFA_SIGNALING_VIA_NFKB | 0.1 | 0.555 | 0.288 | 0.311 | 1.15 | 184 | Bmp2/Cxcl2/Areg/Lif/Il23a/Atf3/Hbegf/Ptx3/Ifit2/Tnfaip6/Serpinb8/Slc2a6/Dusp4/Cxcl1/Gem/Gfpt2/Cxcl10/Klf4/Tnfaip3/Tlr2/Fosb/Nr4a1/Klf6/Trib1/Spsb1/B4galt5/Bhlhe40/Icam1/Fosl1/Klf10/Bcl3/Nr4a3/Tnf/Fosl2/Ifih1/Efna1/Maff/Irs2/Sqstm1/Zbtb10/Per1/Rel/Btg2/Egr1/Nfkbia/Mcl1/Rela/Lamb3/Tiparp/B4galt1/Ldlr/Ccnl1/Irf1/Edn1/Id2/Klf2/Zc3h12a/Birc3/Gadd45b/Cebpd/F3/Nfkbie/Ppp1r15a/Plk2 |
| HALLMARK_E2F_TARGETS | 0.177 | 0.626 | 0.249 | 0.301 | 1.12 | 196 | Spag5/Kif4/Kif2c/Timeless/Kif18b/Hmmr/Ccne1/Wee1/Chek1/Pole/Dscc1/Birc5/Aurkb/Diaph3/Cdkn3/Cit/Rad51c/Hells/Mcm4/Atad2/Trp53/Brca2/Prim2/Racgap1/Prps1/Donson/Ipo7/Gins1/Dclre1b/Espl1/Mlh1/Ubr7/Smc4/Rad21/Mcm3/Cdc20/Pds5b/Cdkn1b/Cdkn1a/Spc25/Orc2/Plk4/Mki67/Lbr/Cdc25b/Cdca8/Pms2/Nup205/Rad50/Tra2b/Jpt1/Pola2/Tmpo/Paics/Aurka/Rad1/Tfrc |
| HALLMARK_INTERFERON_ALPHA_RESPONSE | 0.305 | 0.687 | 0.225 | 0.32 | 1.08 | 91 | Ifit3/Batf2/Ifit2/Ifitm1/Parp14/Tmem140/Cxcl10/Procr/Rsad2/Il4ra/Mx2/Ifih1/Ifi44/Ifi44l/Cmpk2/Helz2/Herc6/Gbp3/Oasl1/Mov10/Irf1/Ddx60/Lgals3bp/Usp18/Tap1/Ifitm3/Gmpr/Tent5a/Plscr1/Cd47/Ripk2/Psmb8/Parp12 |
| HALLMARK_UV_RESPONSE_UP | 0.216 | 0.626 | 0.249 | 0.297 | 1.05 | 149 | Bmp2/Mapk8ip2/Atf3/Dlg4/Ccne1/Lhx2/Ret/Rrad/Cxcl1/Fosb/Nr4a1/Cdkn2b/Icam1/Maoa/Ppat/Gal/Sqstm1/Chka/Btg2/Nfkbia/Fmo1/Hspa13 |
| HALLMARK_G2M_CHECKPOINT | 0.711 | 1 | 0.144 | 0.269 | 1 | 192 | Ttk/Exo1/Kif4/E2f1/Kif2c/Ube2c/Hmmr/Kif11/Pbk/Chek1/Wrn/Pole/Incenp/Ndc80/Kif15/Bub1/Birc5/Aurkb/Kif23/Bcl3/Dbf4/Cdkn3/Nup98/Abl1/Slc38a1/Brca2/Prim2/Racgap1/Ccna2/Dmd/Espl1/E2f3/Tfdp1/Arid4a/Atf5/Smc4/Rad21/Nusap1/Mcm3/Cdc20/Pds5b/Cdkn1b |
| HALLMARK_P53_PATHWAY | 0.913 | 1 | 0.219 | 0.24 | 0.9 | 197 | Bmp2/Lif/Fgf13/Upp1/Atf3/Hbegf/Krt17/Epha2/H1f2/Rrad/Klk8/Klf4/Apaf1/Procr/Cdkn2b/Clca2/Kif13b/Sfn/Acvr1b/Plk3/Ddit3/Rhbdf2/Rad51c/Vdr/Ndrg1/Ak1/Btg2/Phlda3/Slc19a2/Ralgds/Tm4sf1/Trp53/Nupr1/Mapkapk3/Trib3/Vwa5a/Dnttip2/Ddb2/Tgfa/Ppp1r15a/Plk2/Zfp36l1/Rgs16/Pom121/Tap1/Rnf19b/Wwp1 |
| HALLMARK_PROTEIN_SECRETION | 0.906 | 1 | 0.126 | 0.247 | 0.84 | 93 | Rps6ka3/Tspan8/Sspn/Pam/Sgms1/Ica1/Adam10/Stam/Tom1l1/Vps4b/Abca1/Arfgap3/Krt18/Cln5/Dst/Rab9/M6pr/Cltc/Dop1a/Clcn3/Ap3s1/Vps45/Atp6v1b1/Galc/Arfip1/Lman1/Snap23/Stx16/Atp1a1/Sec22b/Snx2/Vamp3/Scamp1/Copb2/Gosr2/Stx12/Gla/Rab5a/Atp7a/Atp6v1h/Rab2a/Mapk1/Anp32e/Gbf1/Arfgef1/Copb1/Rab22a/Arcn1 |
| HALLMARK_MITOTIC_SPINDLE | 0.976 | 1 | 0.225 | 0.22 | 0.81 | 199 | Ttk/Anln/Kif4/Kntc1/Kif2c/Kif11/Dock4/Ect2/Cep192/Incenp/Arhgap29/Ndc80/Kif15/Bub1/Birc5/Kif23/Nedd9/Ccdc88a/Cntrob/Rabgap1/Alms1/Abl1/Cdc42ep1/Brca2/Racgap1/Fgd4/Espl1/Cdc42ep2/Akap13/Arhgap27/Smc4/Ppp4r2/Tubgcp3/Nusap1/Mid1/Itsn1/Ophn1/Sptbn1/Hook3/Cep57/Epb41l2/Ralbp1/Arhgef3/Wasl/Pcnt/Abi1/Bcar1/Epb41/Dlg1/Nck1/Dst/Arhgap5/Sptan1/Csnk1d/Aurka/Rab3gap1/Plekhg2/Pdlim5/Cd2ap/Cdc42ep4/Clip1/Sos1/Abr/Pxn/Ezr/Katnb1/Cdk5rap2/Smc1a |
| HALLMARK_UV_RESPONSE_DN | 1 | 1 | 0.152 | 0.221 | 0.77 | 136 | Amph/Ptgfr/Col3a1/Scn8a/Adora2b/Igfbp5/Mgmt/Bhlhe40/Cacna1a/Rnd3/Pdgfrb/Cdon/Gcnt1/Insig1/Ldlr/Cap2/Tgfbr3/ |

| | | | | | | | |
|---|---|---|---|---|---|---|---|
| | | | | | | | F3/Efemp1/Pex14/Wdr37/Atp2c1/Ica1/Adgrl2/Tfpi/Kit/Cdkn1b/Prkca/Acvr2a/Ptpn21/Rbpms/Nr3c1/ Map2k5/Id1/Add3/Akt3/ Sipa1l1/Vldlr/Runx1/Kalrn/Nipbl/Dlg1/Phf3/Nek7/Bmpr1a/Scaf8/Dab2/Smad3/Fyn/Zmiz1/Mmp16/Fzd2/Pdlim5/Dmac2l/Anxa4 |
| HALLMARK_WNT_BETA_CATENIN_SIGNALING | 0.955 | 1 | 0.088 | 0.244 | 0.72 | 41 | Dkk4/Hey1/Hey2/Wnt5b/Dvl2/Rbpj/Trp53/Fzd1/Notch4/Hdac5 |
| HALLMARK_CHOLESTEROL_HOMEOSTASIS | 0.993 | 1 | 0.111 | 0.209 | 0.67 | 69 | Cxcl16/Atf3/Errfi1/Trp53inp1/Cpeb2/Tnfrsf12a/Niban1/Chka/Acss2/Ldlr/Trib3/Sc5d/Atf5/Plscr1/Stard4 |
| HALLMARK_DNA_REPAIR | 1 | 1 | 0.155 | 0.157 | 0.56 | 145 | Npr2/Pde6g |
| HALLMARK_ADIPOGENESIS | 1 | 1 | 0.01 | -0.151 | -0.42 | 194 | Cyp4b1/Sncg/Adig/C3/Ephx2/Gpx3/Slc66a3/Plin2/Mylk/Cavin2/Cdkn2c/Apoe/Acaa2/Nabp1/Ptger3/ Dhcr7/Mgst3/ Ndufb7/Pfkfb3/Retsat/Lipe/Cd302/Uqcr11/Coq3/Uqcrq/Ndufa5/Slc25a10/Ddt/Stom/Stat5a/Taldo1/Uck1/Acly/Jagn1/ Rab34/Agpat3/Idh1/Uqcr10/Pgm1/Qdpr/Slc19a1/Ak2/Gpam/Dnajc15/Cox7b/Echs1/Cox6a1/Mtarc2/ Ndufab1/Scp2/Etfb/Cmbl/Cox8a |
| HALLMARK_UNFOLDED_PROTEIN_RESPONSE | 1 | 1 | 0.016 | -0.201 | -0.54 | 110 | Psat1/Kdelr3/Fkbp14/Slc1a4/Asns/Eif2ak3/Aldh18a1/Chac1/Rps14/Nabp1/Hyou1/Arxes2/Pdia5/Imp3/Arfgap1/Nhp2/Eef2/Mthfd2/Calr/ H2ax/Lsm4 |
| HALLMARK_OXIDATIVE_PHOSPHORYLATION | 1 | 1 | 0.01 | -0.198 | -0.56 | 194 | Maob/AK157302/Acaa2/Mtrf1/Ndufa1/Mgst3/Ndufb7/Atp5f1e/Got2/Retsat/Nqo2/Cox4i1/Mrps12/Cox7a2l/Uqcr11/ Mrpl11/Ndufb3/Uqcrq/Polr2f/Cpt1a/Cox5b/Timm13/Cyb5a/Ndufa2/Mrps11/Cox6c/Ndufa5/Ndufa7/Atp6v0c/Ndufs8/Atp5mf /Ndufa6/Atp5mc1/Cox15/Pdp1/Idh1/Ndufs6/Uqcr10/Uqcrh/Fxn/Atp5pd/Cyb5r3/Cox11/Ndufc1/Cox7c /Mrpl34/Atp5mc3/ Atp5pf/Cox7b/Atp5me/Echs1/Cox6a1/Ndufb5/Cox7a2/Ndufa3/Cox6b1/Bax/Ndufab1/Timm8b/Etfb/Cox8a/Ndufb8/Slc25a4/Ndufs7/ Atp5f1d |
| HALLMARK_FATTY_ACID_METABOLISM | 0.999 | 1 | 0.014 | -0.215 | -0.58 | 142 | Cd1d2/Aqp7/Inmt/Car4/Aldh3a1/Rdh1 |
| HALLMARK_MYC_TARGETS_V1 | 1 | 1 | 0.01 | -0.218 | -0.61 | 193 | Cad/Myc/Srm/Pabpc4/Mcm5/Ctps1/Rrm1/Rps2/Rpl18/Sf3a1/Odc1/Cdc45/Rpl14/Rps5/Mrpl9/Mrpl23 /Rps6/Eef1b2/ Nme1/Rack1/Hddc2/Got2/Snrpg/Rplp0/Nhp2/Hspe1/Fbl/Rps10/Rps3/Pole3/Psma7/Aimp2/Lsm2/Snrpd1/Eif3b/Pa2g4/ Snrpa/Kars1/Eif2s2/Syncrip/Trim28/Nop16/Phb1/Ube2e1/Rfc4/Usp1/Cdk2/Snrpd3/Ndufab1/Rpl22/Psma2/Cct7/Prdx4/ Mcm7/Exosc7/Rad23b/C1qbp/Npm1/Rnps1/Cct5/H2az1/Pabpc1/Ppia/Mad2l1/Rpl6/Stard7 |
| HALLMARK_HEME_METABOLISM | 0.993 | 1 | 0.011 | -0.242 | -0.67 | 174 | Kel/Ftcd/Abcg2/Gypc/Acp5/Trim10/C3/E2f2/Ctse/AK157302/Aldh1l1/Asns/Cdr2/Alad/Blvrb/Xk/Mocos/Epor/Uros/Htatip2 |
| HALLMARK_ANDROGEN_RESPONSE | 0.937 | 1 | 0.022 | -0.275 | -0.72 | 92 | Inpp4b/Steap4/Azgp1/Gucy1a1/Pmepa1/Adamts1/Ptk2b/Nkx3-1/Hpgd/Mak/Ank/Ccnd1 |
| HALLMARK_BILE_ACID_METABOLISM | 0.891 | 1 | 0.023 | -0.288 | -0.76 | 99 | Dio1/Cyp46a1/Pipox/Aqp9/Ch25h/Sult2b1/Ephx2/Fads2/Slc23a1/Ttr/Soat2/Gc |
| HALLMARK_PANCREAS_BETA_CELLS | 0.741 | 1 | 0.042 | -0.375 | -0.82 | 24 | Chga/Abcc8 |
| HALLMARK_SPERMATOGENESIS | 0.81 | 1 | 0.028 | -0.312 | -0.83 | 108 | Nos1/She/Ddx4/Pcsk1n/Snap91/Slc2a5/Tekt2/Tktl1/Hspa1l/Oaz3/Ncaph/Art3/Sycp1/Nek2/Acrbp/Acrv1/Pebp1/Pomc/Grm8/Pcsk4/ Cdk1/Cst8/Dmc1/Ldhc/Ace/Clgn/Septin4/Slc12a2 |
| HALLMARK_ESTROGEN_RESPONSE_ | 0.6 | 0.9 | 0.0 | -0.34 | -0.95 | 187 | Igfbp4/Abat/Krt15/Fcmr/Fhl2/Tfap2c/Myb/Sult2b1/Gfra1/Inhbb/Cbfa2t3/Cxcl12/Rapgefl1/Pdzk1/Pod |

| | | | | | | | |
|---|---|---|---|---|---|---|---|
| EARLY | 07 | 49 | 39 | | | | xl/Ttc39a/Adcy1/Tpbg/Lad1/ Slc1a4/Celsr2/Myc/Syt12/Rasgrp1/Anxa9/Slc1a1/Tsku/Gja1/Slc22a5/Ccnd1/Clic3/Cyp26b1/Opn3/Igf1r/Tiam1/Blvrb/Zfp185/Slc16a1 |
| HALLMARK_XENOBIOTIC_METABOLISM | 0.5 46 | 0.8 81 | 0.0 44 | -0.354 | -0.98 | 172 | Igfbp4/Crp/Mbl2/Hsd11b1/Aqp9/Xdh/Acox2/Ptgds/Fbp1/Arg2/Itih4/Aldh3a1/Arg1/Slc6a12/Fbln1/Atoh8/Cyp17a1/Ptgr1/Hgfac /Gch1/Kynu/Nqo1/Irf8/Tat/Ces1d/Aox1/Ccl25/Lcat/Tmem97/Acox3/Fmo3/Pycr1/Blvrb/Apoe |
| HALLMARK_GLYCOLYSIS | 0.4 97 | 0.8 34 | 0.0 48 | -0.355 | -1 | 191 | B3gat1/Col5a1/Lct/Ppfia4/Pfkfb1/Hs6st2/Stc1/Cxcr4/Tktl1/Chst2/Cth/Kdelr3/Qsox1/Ak4/Tpbg/Efna3/Isg20/Vcan/Pygl/Eno1b/ Plod1/Nanp/Spag4/Gale/Cdk1 |
| HALLMARK_APICAL_JUNCTION | 0.5 01 | 0.8 34 | 0.0 48 | -0.36 | -1 | 177 | Tnfrsf11b/Rac2/Icam5/Sirpa/Nfasc/Gamt/Col16a1/Cd86/Adam23/Slc30a3/Nexn/Slit2/Pecam1/Cadm2/Gnai1/Syk/Cldn14/ Col17a1/Krt31/Cd34/Mpzl2/Vcan/Nherf4/Adra1b/Fyb1/Ptprc/Ppp2r2c/Nrtn/Itga10/Acta1/Lama3/Irs1/ Traf1/Kcnh2/Nectin3/ Amigo1/Fbn1/Bmp1/Alox8/Nectin1/Nlgn2 |
| HALLMARK_PEROXISOME | 0.4 28 | 0.7 64 | 0.0 59 | -0.393 | -1.03 | 94 | Dio1/Abcc8/Esr2/Cacna1b/Crabp2/Sult2b1/Top2a/Abcb4/Ephx2 |
| HALLMARK_MYC_TARGETS_V2 | 0.4 08 | 0.7 56 | 0.0 64 | -0.415 | -1.04 | 58 | Slc29a2/Map3k6/Myc/Plk1/Srm/Mcm5/Tmem97/Pprc1/Utp20/Rrp12/Hk2/Hspe1/Nop2/Grwd1/Bysl/Aimp2/Pus1/Noc4l/ Ndufaf4/Pa2g4/Slc19a1/Nop16/Phb1/Imp4 |
| HALLMARK_HYPOXIA | 0.4 04 | 0.7 56 | 0.0 58 | -0.371 | -1.04 | 189 | Pkp1/Plaur/Hs3st1/S100a4/Stbd1/Col5a1/Ppfia4/Slc2a3/Ets1/Fbp1/Stc1/Slc2a5/Bgn/Cxcr4/Tktl1/Sult2b1/Large1/Chst2/ Chst3/Pygm/Kdelr3/Ak4/Tpbg/Efna3/Isg20/Plin2 |
| HALLMARK_PI3K_AKT_MTOR_SIGNALING | 0.3 76 | 0.7 52 | 0.0 64 | -0.4 | -1.06 | 99 | Sla/Prkcb/Il2rg/Cxcr4/Mapk10 |
| HALLMARK_MYOGENESIS | 0.3 02 | 0.6 87 | 0.0 72 | -0.394 | -1.09 | 165 | Tnni1/Mef2c/Lsp1/Ppfia4/Kcnh1/Nos1/Casq2/Apod/Col6a3/Vipr1/Tnnt2/Sphk1/Des/Sod3/Fhl1/Tpm2/Sparc/Ncam1/ Casq1/Myoz1/Large1/Mb/Pygm/Gpx3/Schip1/Sh3bgr/Cacng1/Mylk/Notch1/Lama2/Igfbp7/Efs/Nqo1/ Acta1/Ckm/Kifc3/ Ldb3/Kcnh2/Col1a1/Fxyd1 |
| HALLMARK_NOTCH_SIGNALING | 0.3 39 | 0.7 05 | 0.0 78 | -0.474 | -1.09 | 32 | Wnt2/Lfng/Dtx4/Wnt5a/Notch3/Dtx1/Notch1/Notch2/Ccnd1 |
| HALLMARK_ANGIOGENESIS | 0.3 16 | 0.6 87 | 0.0 82 | -0.493 | -1.11 | 30 | S100a4/Cxcl5/Apoh/Stc1/Prg2/Ccnd2/Vcan/Pf4 |
| HALLMARK_REACTIVE_OXYGEN_SPECIES_PATHWAY | 0.3 06 | 0.6 87 | 0.0 8 | -0.464 | -1.13 | 46 | Lsp1/Hhex/Gpx3/Nqo1 |
| HALLMARK_COAGULATION | 0.2 42 | 0.6 37 | 0.0 87 | -0.425 | -1.13 | 110 | Plek/Gda/Cpn1/Mbl2/Fga/C1qa/Cpb2/Mep1a/Acox2/Proc/Pecam1/P2ry1/Sparc/C1s1/Mmp8/Rgn/Htra1/Mmp7/C3/Fgg/ Ctse/Pf4/Cfi/F8/Trf |
| HALLMARK_ESTROGEN_RESPONSE_LATE | 0.2 07 | 0.6 26 | 0.0 92 | -0.407 | -1.13 | 180 | Th/Igfbp4/Cacna2d2/Batf/Gjb3/Acox2/Stil/S100a9/Ltf/Tfap2c/Myb/Sult2b1/Gper1/Prlr/Fgfr3/Large1/ Cxcl12/Rapgefl1/ Chst8/Pdzk1/Top2a/Tpbg/Isg20/Slc1a4/Celsr2/Ass1/Anxa9/Igsf1/Slc22a5/Gale/Btg3/Ccnd1/Clic3/Cyp26b1/Opn3/Atp2b4/ Cpe/Tiam1/Blvrb/Scube2/Slc16a1 |
| HALLMARK_MTORC1_SIGNALING | 0.2 02 | 0.6 26 | 0.0 93 | -0.404 | -1.13 | 196 | Sla/Coro1a/Itgb2/Fgl2/Slc2a3/Sytl2/Ccnf/Stc1/Cxcr4/Cfp/Ctsc/Cth/Psat1/Phgdh/Ak4/Psph/Slc1a4/Plk1/Fads2/Eno1b/ Asns/Sdf2l1/Tmem97/Fkbp2/Tm7sf2/Cdc25a |
| HALLMARK_APOPTOSIS | 0.1 99 | 0.6 26 | 0.0 96 | -0.418 | -1.14 | 151 | Il1b/Cd2/Cd69/Ereg/Cd38/Casp1/Gucy2e/Plcb2/Gna15/Timp2/Plppr4/Bgn/Brca1/Fez1/Top2a/Cth/Ccnd2/Gpx3/Cd14/Isg20 |
| HALLMARK_IL6_JAK_STAT3_SIGNALIN | 0.2 | 0.6 | 0.0 | -0.448 | -1.16 | 75 | Il1b/Il2rg/Itga4/Cd38/Il2ra/Pik3r5/Ltb/Il12rb1/Il7/Cntfr/Acvrl1/Cd14/Pf4 |

| | | | | | | | |
|---|---|---|---|---|---|---|---|
| G | 25 | 26 | 93 | | | | |
| HALLMARK_IL2_STAT5_SIGNALING | 0.157 | 0.626 | 0.109 | -0.414 | -1.16 | 188 | Il10ra/Sell/Adam19/Cd79b/Cd48/Fgl2/Il2ra/Rhoh/Gpr65/Slc2a3/She/Cd83/Capn3/Ltb/Batf/Cd86/Slc29a2/Selp/Drc1/Tnfrsf8/Tnfrsf9/Itgae/Tnfrsf18/Ccnd2/Col6a1 |
| HALLMARK_INTERFERON_GAMMA_RESPONSE | 0.12 | 0.602 | 0.127 | -0.427 | -1.19 | 176 | Il10ra/H2-Aa/Gpr18/Bank1/Ccl5/Cd69/Stat4/St8sia4/Cd38/Casp1/Fgl2/Itgb7/Slamf7/Cd74/Marchf1/Cd86/Selp/Il7/C1s1/Lysmd2 |
| HALLMARK_APICAL_SURFACE | 0.182 | 0.626 | 0.113 | -0.514 | -1.21 | 40 | Il2rg/Plaur/Efna5/Brca1/Lypd3/Gas1/Slc22a12/Sulf2/Pkhd1 |
| HALLMARK_EPITHELIAL_MESENCHYMAL_TRANSITION | 0.08 | 0.497 | 0.161 | -0.447 | -1.23 | 163 | Matn2/Igfbp4/Plaur/Acta2/Fmod/Tnfrsf11b/Snai2/Col5a1/Vim/Emp3/Nid2/Igfbp2/Cxcl5/Col6a3/Scg2/Sfrp1/Col16a1/Slit2/Bgn/Thbs2/Tpm2/Serpine2/Pthlh/Sparc/Mgp/Gas1/Cxcl12/Cdh2/Wnt5a/Cthrc1/Htra1/Fbln1/Slit3/Qsox1/Inhba/Pmepa1/Vcan/Col8a2/Magee1/Mylk/Lama2/Basp1/Plod1/Fstl3/Col5a3/Nnmt/Lama3/Postn/Il15/Gja1/Col1a1/Fbn1/Notch2/Bmp1/Fbln5/Tagln |
| HALLMARK_KRAS_SIGNALING_DN | 0.047 | 0.389 | 0.217 | -0.475 | -1.29 | 139 | Pkp1/Dlk2/Capn9/Adra2c/Lfng/Igfbp2/Nos1/Kcnd1/Krt15/Tenm2/Kcne2/Tshb/Lgals7/Gamt/Pnmt/Cd80/Edar/Gdnf/Slc30a3/Slc16a7/Smpx/Krt5/Lypd3/Nrip2/Ntf3/Fgfr3/Itgb1bp2/Cntfr/Chst2/Tff2/Cldn16/Egf/Kcnn1/Celsr2/Tfap2b/Cacng1/Irs4/Itih3/Atp4a/Clstn3/Ckm |
| HALLMARK_HEDGEHOG_SIGNALING | 0.065 | 0.467 | 0.2 | -0.588 | -1.36 | 33 | Nrp2/L1cam/Shh/Nrcam/Scg2/Cdk5r1/Slit1/Cntfr |
| HALLMARK_KRAS_SIGNALING_UP | 0.01 | 0.099 | 0.381 | -0.493 | -1.37 | 177 | Ctss/Il10ra/Il1b/Il2rg/Itgb2/Kcnn4/Laptm5/Cd37/Plaur/Ereg/Lcp1/Tspan7/Itgbl1/Dock2/F13a1/Hsd11b1/Reln/Apod/Snap25/Pcsk1n/Snap91/Tnnt2/Ets1/Gucy1a1/Cxcr4/Pecam1/Il33/Prdm1/Clec4a3/Gypc/Map4k1/Pigr/Ikzf1/Arg1/Igf2/Spon1/Gpnmb/Epb41l3/Gprc5b/Ccnd2/Mmd/Inhba |
| HALLMARK_COMPLEMENT | 0.003 | 0.043 | 0.432 | -0.516 | -1.42 | 157 | Plek/Ctss/Gng2/Ccl5/Plaur/Pik3cg/Casp1/L3mbtl4/C1qa/Pik3r5/Timp2/Dock10/Pla2g7/Cdk5r1/S100a9/Ltf/Klk1/C1s1/Kcnip3/Mmp8/Gngt2/Ctsc/C3/Dgkg/Kcnip2/Was/F8/Hpcal4/Rasgrp1/F5/Kynu/Phex/Ctsh |
| HALLMARK_INFLAMMATORY_RESPONSE | 0.003 | 0.043 | 0.432 | -0.517 | -1.43 | 164 | Il10ra/Sell/Il1b/Gpr132/Gpr183/Cd48/Ccl5/Plaur/Cd69/Ereg/Kcna3/Lpar1/Slc28a2/Slc4a4/Gna15/Emp3/Tacr3/Rgs1/Ros1/Cxcl5/Pik3r5/Tnfsf15/Aqp9/Cybb/Nod2/Mep1a/Ccl22/Tnfsf9/Sphk1/Pcdh7/Nmur1/Nlrp3/Tnfrsf9/Chst2 |
| HALLMARK_ALLOGRAFT_REJECTION | 0 | 0 | 0.734 | -0.645 | -1.78 | 162 | Ctss/H2-Ob/Spi1/Prkcb/Dyrk3/Il1b/Il2rg/Itgb2/H2-Aa/Ccr5/Hcls1/H2-DMb2/Ccl5/Ncf4/Cd2/Ereg/Ly86/Stat4/St8sia4/Prkcg/Il12a/Il2ra/Igsf6/Gpr65/Mbl2/Ccr2/Fgr/Cd1d2/Crtam/Cd74/Srgn/Ltb/Cd80/Il12rb1/Ets1/Cd86/Ccl22/Map4k1/Brca1/Il7/Inhbb/Cd247/Nlrp3/Cd4/Cfp/Ccnd2/Inhba/Was/Cd79a/Fcgr2b/Pf4/Rpl3l/Fyb1/Ptprc/H2-DMa/Hdac9 |
| | | | | | | | |

**Supplementary Table 1. Gene Set Enrichment Analysis (GSEA) of differentially expressed genes across epididymal regions.** Detailed results of the GSEA performed using the MSigDB Hallmark gene set collection.
